# HNRNPH1 drives glioblastoma progression by regulating the splicing of cell cycle genes

**DOI:** 10.1038/s41419-026-08576-6

**Published:** 2026-03-24

**Authors:** Genaro R. Villa, Paolo Alimonti, Joseph S. Toker, Raziye Piranlioglu, Mikayla A. Karkoski, Debora Mazzetti, Reda Ben Mrid, Sara El Guendouzi, Alexa Lauinger, Andrew N. Chiocca, Rachid El Fatimy, E. Antonio Chiocca, Marco Mineo

**Affiliations:** 1 Harvey W. Cushing Neuro-oncology Laboratories, Department of Neurosurgery, Brigham and Women’s Hospital and Harvard Medical School, Boston, MA USA; 2 Harvard Medical School, Boston, MA USA; 3 Faculty of Medical Sciences, UM6P Hospitals, Mohammed VI Polytechnic University, Benguerir, Morocco; 4 Carle Illinois College of Medicine, Urbana, IL USA

**Keywords:** CNS cancer, RNA splicing

## Abstract

Although glioblastoma (GBM) harbors multiple genetic abnormalities leading to cell cycle deregulation, a functional mitotic checkpoint is essential to prevent mitotic catastrophe and tumor cell death. Here, we identify the RNA-binding protein HNRNPH1 as a key post-transcriptional modulator of G2/M checkpoint-associated genes in GBM. HNRNPH1 is overexpressed in malignant cells, especially in the neural- and oligodendrocyte-progenitor-like state, and its expression levels are higher in non-hypoxic regions of the tumor. Knocking out HNRNPH1 causes aberrant splicing and downregulation of several genes involved in cell division. These molecular alterations are associated with G2/M cell cycle arrest, reduced cell proliferation, abnormal cell morphology, and increased nuclear fragmentation. Silencing HNRNPH1 in vivo inhibits the tumor growth of patient-derived GBM cell-originated intracranial xenografts and has significant survival benefits. Together, our results show the critical importance of HNRNPH1 in cell cycle progression and tumor growth, potentially impacting the development of novel strategies to treat GBM.

## Introduction

Glioblastoma (GBM) is the most aggressive and lethal primary brain tumor in adults, characterized by rapid proliferation and resistance to standard therapies [1]. The uncontrolled growth of GBM is driven by key genetic alterations, including TP53 mutations, PTEN loss, CDKN2A deletion, and EGFR amplification, each of which perturbs cell cycle control and plays a crucial role in promoting continuous cell division [2–5]. While these mutations give GBM cells a selective advantage, tumor survival also depends on the integrity of mitotic processes [6]. Mitotic checkpoints function to prevent errors during cell division, and their disruption can lead to mitotic catastrophe, a form of cell death triggered by defective mitosis [7, 8]. Identifying pathways that regulate mitosis in GBM could reveal novel therapeutic strategies to induce mitotic catastrophe and inhibit tumor progression. A fundamental question remains: which molecular mechanisms enable GBM cells to sustain mitotic integrity and evade catastrophic failure?

RNA splicing, particularly alternative splicing (AS), is a critical process that diversifies the transcriptome and modulates gene function [9]. Widespread splicing dysregulation is recognized as a hallmark of cancer, influencing cell cycle progression, apoptosis, and stress responses [10, 11]. Increasing evidence has suggested that AS dysregulation in cancer is primarily due to alterations in the expression of RNA-binding proteins (RBPs) [12, 13]. Notably, the rate of recurrent AS events in cancer-driver genes exceeded their mutational rate and was associated with a worse prognosis in GBM [14]. Therefore, a deeper understanding of how RBPs regulate AS in GBM may yield an array of new actionable therapeutic targets.

The heterogeneous nuclear ribonucleoprotein (HNRNP) family of RBPs are critical regulators of RNA metabolism [15], and their aberrant expression is implicated in several disease states, including Ewing Sarcoma [16], chronic myeloid leukemia [17], prostate cancer [18], and neuronal-related disorders [19, 20]. Many HNRNPs share general features but differ in domain composition and functional properties. They can present up to four RNA-binding domains, also called “RNA recognition motifs” (RRM), and different auxiliary domains that can undergo post-translational modification and possess various functions, including protein-protein interaction and nuclear localization [15, 21]. Among the several different HNRNP family members, HNRNPH1 is characterized by a non-conserved structure of the RRMs described as quasi-RRMs (qRRMs) [22]. These qRRMs interact with G-rich RNA sequences that regulate the splicing of various pre-mRNAs [23, 24]. HNRNPH1 has been shown to function as a critical regulator of genes encoding proteins that promote cancer initiation and progression [18, 25]. We have recently identified the transcripts that HNRNPH1 binds in tumor cells [26]. However, the role of HNRNPH1 in GBM development remains poorly understood.

Herein, we show that HNRNPH1 is a key player in GBM pathophysiology. By combining single-cell and spatial transcriptomic data, we find that HNRNPH1 is overexpressed in GBM tumor cells, and its expression is associated with specific cellular states and tumor regions. By ensuring the proper splicing of essential transcripts for mitotic division, HNRNPH1 maintains mitotic stability, sustains proliferation, and promotes rapid GBM tumor growth. Taken together, our data reveal that HNRNPH1 is a critical mediator of gene regulation, suggesting the development of HNRNPH1-directed therapy as a potential novel strategy in GBM.

## Materials and Methods

### Human specimens

Tumor tissue samples were obtained with the consent of the participants, as approved by the Institutional Review Board (IRB) at the Dana-Farber Cancer Institute. Patient samples were processed for the extraction of total RNA.

### Data collection

Gene expression data for HNRNP H family members and analysis of HNRNPH1 expression correlation with cell cycle genes were downloaded from The Georgetown Database of Cancer (G-DOC) at https://gdochub.georgetown.edu/ using the TCGA Glioblastoma (GBM) dataset. Gene expression analysis in the different anatomical regions of GBM was performed using the Ivy Glioblastoma Atlas Project (https://glioblastoma.alleninstitute.org/). Single-cell RNA sequencing data and spatial transcriptomics data were obtained from Gene Expression Omnibus (GEO) (GSE131928; GSE237183).

### Cell culture

Patient-derived primary GBM cells (G62, BT139, and BT245 cell lines) were generated as previously described [26] and cultured as neurospheres in stem cell conditions using Neurobasal (Thermo Fisher Scientific, Waltham, MA, USA) supplemented with Glutamine (Thermo Fisher Scientific), B27 (Thermo Fisher Scientific), 20 ng/ml epidermal growth factor (EGF) and fibroblast growth factor (FGF)-2 (PrepoTech/Thermo Fisher Scientific, Waltham, MA, USA). U87 and HEK-293 cells were purchased from the American Type Culture Collection (ATCC, Manassas, VA, USA) and cultured in DMEM (Thermo Fisher Scientific) supplemented with 10% fetal bovine serum (FBS, Sigma-Aldrich, St. Louis, MO, USA). U87 and G62 cells were recently authenticated by STR profiling. All cell lines were routinely tested for mycoplasma contamination. *HNRNPH1* knockout in U87 cells was performed by transfecting 3 pmol/μl of a three synthetic gRNA system from Synthego (Redwood City, CA, USA) (Gene Knockout Kit v2 - human - HNRNPH1) and 3 pmol/μl TrueCut Cas9 Protein v2 (Thermo Fisher Scientific) for 24-well plate using Lipofectamine CRISPRMAX (Thermo Fisher Scientific). *HNRNPH1* knockout in G62 cells was performed by nucleofecting 30 pmol/μl of sgRNAs and 20 pmol/μl of TrueCut Cas9 Protein v2 using P3 Primary Cell 4D-Nucleofector X Kit L (Lonza, Basel, Switzerland) in a 4D-Nucleofector X unit (Lonza). Rescue experiments were performed by transfecting/nucleofecting U87 or G62 cells with the human HNRNPH1 coding sequence cloned into pcDNA3.1/CT-GFP-TOPO (Thermo Fisher Scientific) 3 days post sgRNA delivery. The primers used for HNRNPH1 amplification are listed in Supplementary Table 1. HNRNPH1 knockdown in G62 cells was performed by transfecting 50 pmol/well of ON-TARGETplus Human HNRNPH1 siRNA SMARTpool (Dharmacon, Lafayette, CO, USA) for 6-well plates using Lipofectamine RNAiMAX (Thermo Fisher Scientific). Deletion of the HNRNPH1 binding site in *UHRF2* intron 12 was obtained by transfecting HEK293 cells with four synthetic gRNAs: sgRNA#1 AAUUUAGGUAUAGGAGGCCC, sgRNA#2 GGACAAAUUCUAGGGUAUAC, sgRNA#3 GGGAGGUGGCUUUAUAAGAG, and sgRNA#4 UCCUAGAUGUCAGACUGCCC. Cell growth analysis was performed by plating 8000 cells/well in a 96-well plate. Cell proliferation was analyzed using PrestoBlue reagent (Thermo Fisher Scientific) following the manufacturer’s instructions. Limiting dilution assay was performed by plating cells in a 96-well plate at different concentrations (from 1 to 1000 cells per well) in 0.1 mL of supplemented Neurobasal medium. Cultures were left undisturbed for 7 days. After incubation, the percentage of wells not containing spheres for each concentration was calculated and plotted against the number of cells per well.

### Immunohistochemistry (IHC)

The tissue microarray was obtained from US Biomax (Derwood, MD, USA). Paraffin-embedded tissues were baked at 60 °C for 1 h and deparaffinized in two washes of 100% xylene for 10 min. Tissues were rehydrated in graded ethanol solutions, followed by two washes in water. Antigen retrieval was performed by incubating the slide in boiling 10 mM sodium citrate buffer pH 6.0. Sections were then incubated in 3% hydrogen peroxide for 10 min, followed by incubation for 1 h in blocking solution (TBST containing 2% normal goat serum). Tissue sections were incubated with primary anti-HNRNPH1 antibody (A300-511A, Bethyl Laboratories, Montgomery, TX, USA) in TBST containing 2% normal goat serum for 1 h at RT. After washing, sections were incubated with horseradish peroxidase-conjugated anti-rabbit secondary antibody (Cell Signaling, Danvers, MA, USA) in TBST for 30 min at RT. Sections were stained using DAB substrate (Thermo Fisher Scientific) and counterstained in hematoxylin.

### Analysis of single-cell RNA sequencing data

For the GBM single-cell RNA sequencing dataset, processed and normalized transcripts per million (TPM) counts from 28 GBM tumors (GEO: GSE131928) were downloaded from GEO, log-transformed, and passed into a Seurat object in R. Relevant clinical, cluster ID, and cell module metadata were also downloaded from GEO and the Broad Institute Single-Cell Portal. Downstream clustering analyses were performed using the Seurat RunPCA, FindNeighbors, FindClusters, and RunUMAP functions with the ‘dims’ variable set equal to ‘1:10’. T cell, macrophage, and oligodendrocyte clusters were defined using the authors’ cell annotation metadata. AC-like, MES-like, OPC-like, and NPC-like tumor cells were defined as cells present in the bottom left, bottom right, top left, and top right quadrants, respectively, of the meta-module plot generated by the authors.

### Analysis of spatial transcriptomics data

Visium count, image, and metaprogram assignment data for 26 GBM sections (GSE237183) were downloaded from GitHub (https://github.com/tiroshlab/Spatial_Glioma). Count and image files for each section were read into R using the Read10X_Image and Load10X_Spatial functions from Seurat. For each section, counts were normalized with the NormalizeData function and scaled with the ScaleData function, while variable features were detected using the FindVariableFeatures function, all using default parameters. The RunPCA function was subsequently applied to each Seurat object, with the features variable set equal to the VariableFeatures. For visualizing co-expression, smoothed feature scores for *HNRNPH1*, *VEGFA*, *AURKB*, *ESPL1*, *MYBL2*, *PRC1*, and *CCNF* were generated using the SmoothKNN function from UCell, with the reduction variable set equal to “pca” and k set equal to 20.

### Quantitative Real-Time PCR analysis

Total RNA was extracted using TRIzol (Thermo Fisher Scientific), reverse transcribed using iScript cDNA Synthesis Kit (BioRad, Hercules, CA, USA), and quantitative real-time PCR was performed using SYBR Green Master Mix (Applied Biosystems, Waltham, MA, USA). 18S expression level was used as control. The primers used throughout the study are listed in Supplementary Table 1.

### Minigene cloning

Genomic DNA was extracted from cell cultures using the Genomic DNA Purification Kit (Thermo Fisher Scientific) to generate amplicons corresponding to the sequence spanning exon1-2 of the *AURKB* gene. PCR products were cloned in pcDNA3.1/CT-GFP-TOPO (Thermo Fisher Scientific) to generate pcDNA3.1-AURKB-GFP minigene. The intronic G-rich sequence in the AURKB minigene was mutated using the Q5 Site-Directed Mutagenesis Kit (New England Biolabs, Ipswich, MA, USA) following the manufacturer’s instructions. WT and mutated minigene were transfected into the cells using Lipofectamine 2000 (Thermo Fisher Scientific). The primers used for minigene amplification and site-directed mutagenesis are listed in Supplementary Table 1.

### RNA-Seq and analysis of RNA-Seq data

RNA was extracted from *HNRNPH1*-knockout U87 cells and *HNRNPH1*-knockdown G62 cells using TRIzol (Thermo Fisher Scientific). 1 µg of total RNA was used, and RNA libraries were prepared using the TruSeq Stranded Total RNA LT Sample Prep kit (Illumina, San Diego, CA, USA) for U87 and Illumina Stranded mRNA kit (Illumina) for G62. Paired-end reads were sequenced on an Illumina NovaSeq X Plus System (Illumina) to achieve at least 40 million reads per sample. The Trimmomatic program was used to remove adapter sequences and bases with base quality lower than three from the ends. Also, using sliding window method, bases of reads that did not qualify for window size 4, and mean quality 15 were trimmed. Afterwards, reads with lengths shorter than 36 bp were dropped to produce trimmed data. Trimmed reads were aligned to the human genome from the UCSC hg38 using HISAT2. DESeq2 was used to normalize expression counts and to obtain differentially expressed genes between cohorts.

### Pathway enrichment analysis

Pathway enrichment analysis was conducted using Gene Set Enrichment Analysis (GSEA) software version 4.1.0. Analyses in GSEA were performed on the normalized counts. The analysis used the gene set database h.all.v2022.2.Hs.symbols.gmt (Hallmarks) with number of permutations set at 1000, collapse/remap to gene symbol = collapse, permutation type = gene_set, enrichment statistic = weighted, metric for ranking genes = Signal2Noise, gene list sorting mode = real, gene list ordering mode = descending, max size: exclude larger sets = 500, min size: exclude smaller sets = 15.

### Immunoblot analysis and antibodies

Immunoblotting was performed as previously described [27]. The following antibodies were used: anti-Aurora B, anti-b-Myb, anti-Cyclin F, anti-PRC1, anti-UBE2S, anti-Stathmin, anti-UHRF2, anti-Cyclin D1 and anti-β-Actin (3094, 33056, 81045, 3639, 13655, 49114, 55506, and 3700, respectively, Cell Signaling Technology); anti-hnRNP-H (A300-511A, Bethyl Laboratories); anti-ESPL1 (H00009700-M01, Abnova, Taipei City, Taiwan).

### UV-crosslink RNA immunoprecipitation

Cells were UV irradiated at 400 mJ/cm2 and lysed in modified RIPA buffer (50 mM Tris, 150 mM NaCl, 4 mM EDTA, 1% NP-40, 0.1% Na-deoxycholate, 0.5 mM DTT, 100U/ml RNasin, protease and phosphatase inhibitors). Samples were sonicated with microtip, 5 watts power (25% duty) for 60 seconds total in pulses of 1 s on followed by 3 s off. DNA was digested by incubating samples for 15 min at 37 °C in 1X DNase salt solution (2.5 mM MgCl2, 0.5 mM CaCl2) with 30 U TurboDNase. EDTA was added to the samples to a final concentration of 4 mM, and samples were centrifuged at 16,000 x g for 10 min. Nuclear extracts were precleared with Protein A/G Plus Agarose beads (Thermo Fisher Scientific) and incubated with primary antibody (anti-hnRNP-H) or rabbit IgG control (Bethyl Laboratories) overnight at 4 °C. Protein/RNA complexes were precipitated using Protein A/G Plus Agarose beads. Beads were washed and incubated with Proteinase K (Thermo Fisher Scientific) and RNA was extracted using TRIzol.

### Analysis of alternative splicing (AS)

Raw sequencing reads from the U87 RNA sequencing underwent preprocessing with Trimmomatic-0.39 to remove adapter sequences, while FastQC v0.11.9 was used to assess read quality. Paired-end reads were then aligned to the GRCh38. p14 human reference genome (GRCh38.primary_assembly.genome.fa) using STAR-2.7.11b in two-pass mode (10.1093/bioinformatics/btv642) to improve splice junction detection. For first-pass alignment, GENCODE basic gene annotation (gencode.v47.annotation.gtf) was applied. Low-quality reads and those mapping to 10 or more locations were filtered out using Samtools v1.9. Differential AS events between conditions were identified using rMATS-turbo v4.3.0 (10.1038/s41596-023-00944-2), which employs a modified generalized linear mixed model to detect splicing variations. The analysis was performed using BAM files from two groups (U87-Control and U87-KO), applying both junction count exon count (JCEC) and junction count (JC) approaches. AS events were filtered based on an absolute Inclusion Level Difference (|ΔPSI | ) > 0.05 and a false discovery rate (FDR) < 0.05. Percent Spliced In (PSI) values were quantified by running rMATS in turbo mode.

### Flow cytometry

Cells were harvested 6 days post-sgRNA transfection and resuspended in ice-cold Phosphate Buffered Saline. Fixation was performed by adding ice-cold 95% ethanol, followed by incubation on ice for 1 h. After fixation, cells were rehydrated in Phosphate Buffered Saline and treated with RNase A solution (1 mg/ml) for 15 min at 37 °C. Propidium iodide was added at 50 µg/ml, and flow cytometry was performed on a BD LSR II (BD Biosciences, San Jose, CA, USA). Data were analyzed using FlowJo software (version 10).

### Immunofluorescence

Cells were treated overnight with nocodazole. Cells were fixed with 4% paraformaldehyde for 10 min, permeabilized for 5 min with 0.3% Triton-X100, blocked with 2% BSA for 1 h. Cells were incubated with alpha-tubulin (2144, Cell Signaling Technology), Aurora B antibody (3094, Cell Signaling Technology) or ESPL1 antibody (H00009700-M01, Abnova) overnight, after which they were incubated for 1 h at RT with secondary anti-rabbit AlexaFluor 594 (Jackson ImmunoResearch, West Grove, PA, USA), followed by incubation with Hoechst 33342 (Thermo Fisher Scientific) for 30 min. Confocal images were acquired with ZEN software on a Zeiss LSM 710 Confocal system (Carl Zeiss Inc., Oberkochen, Germany).

Brain sections were permeabilized for 10 min with 0.25% Triton-X100 and blocked with 1% donkey serum for 1 h. Brain sections were incubated overnight with anti-hnRNP-H (A300-511A, Bethyl Laboratories), after which they were incubated for 1 h at RT with secondary anti-rabbit AlexaFluor 594 (Jackson ImmunoResearch) and Hoechst 33342 (Thermo Fisher Scientific). Images were acquired with a Nikon Eclipse Ti (Nikon Instruments Inc., Melville, NY, USA).

### In vivo studies

Mice studies were conducted in accordance with protocols approved by the Institutional Animal Care and Use Committee (IACUC). The maximal tumor size permitted by the IACUC (5 mm) was not exceeded in this study. Control and *HNRNPH1*-knockout cells (5000 cells) were stereotactically injected into the brains of 6- to 8-week-old female nude mice (Jackson, Bar Harbor, ME), with 11 mice per group. On day 11 post-injection, three mice per group were sacrificed, perfused with phosphate buffer saline, and fixed in formalin. Brains were sectioned and stained by incubation with Hoechst 33342 (Thermo Fisher Scientific) for 30 min. Brains were imaged using a Nikon Eclipse Ti microscope (Nikon Instruments Inc.), and tumor volumes were analyzed using the NIS-Elements software. Mouse survival (*n* = 8 per group) was followed until the predetermined IACUC-approved endpoint was reached. The animal sample size was estimated based on previous studies, which showed that eight mice per group provided sufficient statistical power to identify the specified improvement.

### Statistical analysis

Data are expressed as mean ± SD of three replicates. All data are representative of at least two independent experiments. Statistical analyses were performed using the two-way ANOVA or the unpaired two-tailed Student’s t-test from GraphPad Prism software. Differences were considered statistically significant at *P* < 0.05. For differential gene expression of the RNA sequencing data, significant genes were detected using DESeq-generated Benjamini-Hochberg (BH)-adjusted p-values (FDR) that were less than 0.01. In the GSEA analysis, enriched gene sets with a false discovery rate (FDR) q-value less than 0.25 were considered significant. Statistical comparison of gene expression in TCGA data and in the single-cell RNA sequencing study was performed using the Wilcoxon test and the stat_compare_means function from the ggpubr R package. Overall survival analysis was performed using GraphPad Prism version 10.4.1. Statistical comparison of the survival distribution of the two groups was performed using the log-rank test. No blinding or randomization was used. All samples and animals were included in the analysis. Variances were compatible between groups.

## Results

### HNRNPH1 is overexpressed in GBM

**Fig. 1:**
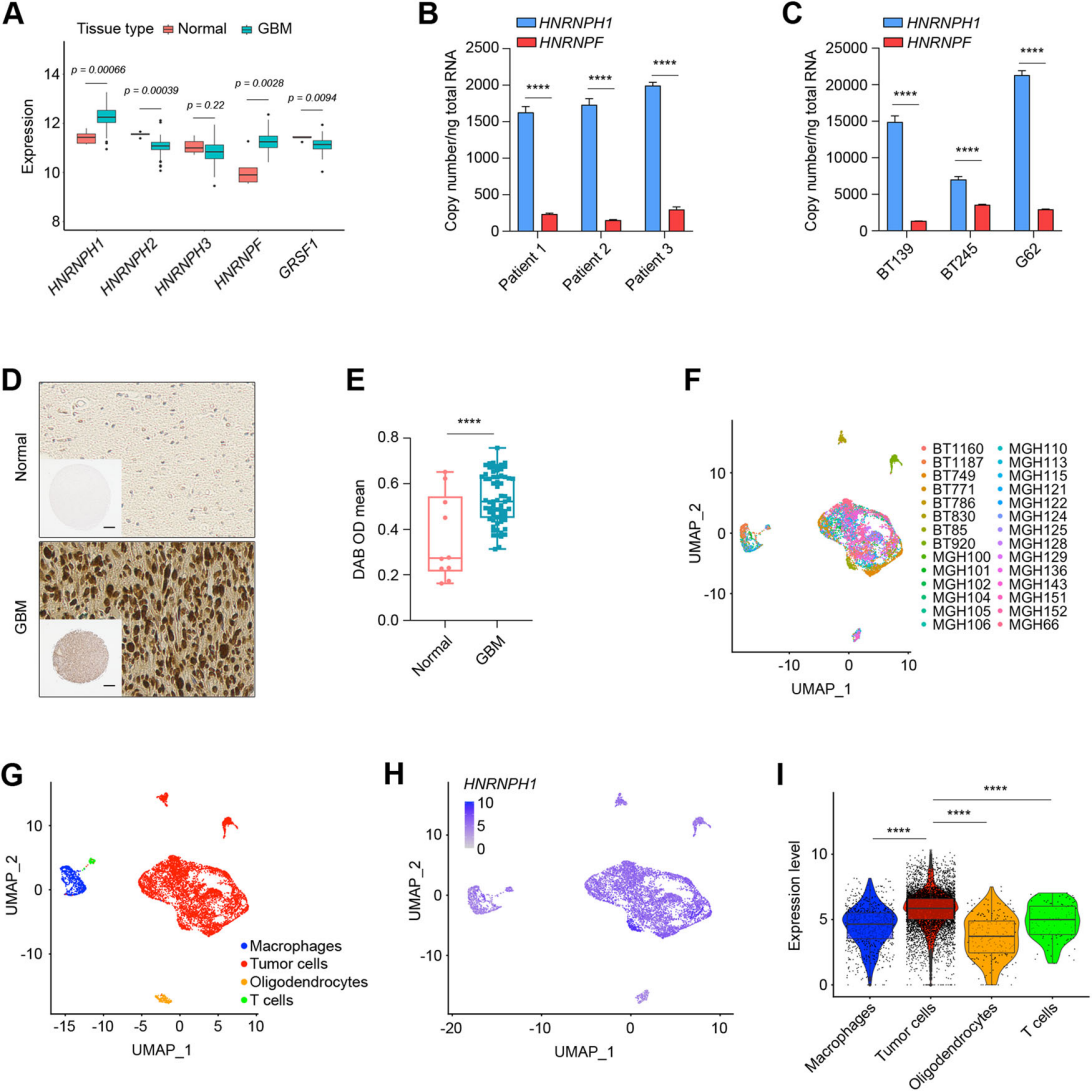
HNRNPH1 is overexpressed in GBM. **A** Analysis of *HNRNPH1*, *HNRNPH2*, *HNRNPH3*, *HNRNPF*, and *GRSF1* expression in The Cancer Genome Atlas (TCGA) GBM dataset. **B**, **C** qRT-PCR analysis of *HNRNPH1* and *HNRNPF* copy numbers in three GBM patient samples (**B**) and three different patient-derived GBM cell lines (**C**). Data shown as mean ± SD of three replicates. Data were analyzed by unpaired t-test: ****p < 0.0001. **D** Representative images of immunohistochemical staining of HNRNPH1 in normal brain (top) and GBM (bottom) tissues from a tissue microarray. Scale bar in low magnification, 300 µm. **E** Quantification of HNRNPH1 expression levels in normal brain and GBM tissues from the tissue microarray using QuPath software. **F** Uniform manifold approximation and projection (UMAP) visualization of 28 GBM patients colored based on patient ID (*n* = 7930). **G** UMAP visualization of single cells colored based on the expression of marker genes for macrophages (blue), tumor cells (red), oligodendrocytes (orange), or T cells (green). **H** UMAP visualization of *HNRNPH1* expression in single cells. **I** Box plot of *HNRNPH1* expression (normalized counts) between macrophages (blue), tumor cells (red), oligodendrocytes (orange), or T cells (green). Data were analyzed by Wilcoxon test: ****p < 0.0001.

The members of the HNRNP H family include five proteins: HNRNPH1, HNRNPH2, HNRNPH3, HNRNPF, and GRSF1 [22]. To investigate the expression of HNRNP H family members in GBM, we first analyzed the TCGA data of 154 GBMs and five normal brains. We found significant upregulation of *HNRNPH1* and *HNRNPF* in GBM compared to normal brains (Fig. 1A). GBM also showed downregulation of *HNRNPH2* and *GRSF1* but no significant changes in *HNRNPH3* levels (Fig. 1A). Since *HNRNPH1* and *HNRNPF* were the only two family members that showed upregulation, we next analyzed their expression in GBM patient specimens and patient-derived GBM cells (PDGCs) by qPCR. We found that both GBM specimens (Fig. 1B) and PDGCs (Fig. 1C) had significantly higher levels of *HNRNPH1* than *HNRNPF*. Given the growing evidence of HNRNPH1’s contribution to the expression of cancer-specific transcripts, we looked more closely at the role of HNRNPH1 in GBM. Immunohistochemical (IHC) analysis of a tissue microarray containing 35 pairs of GBMs and five pairs of normal brain tissues showed positive HNRNPH1 staining in the nucleus of cells, consistent with previous studies showing HNRNPH1 as a mostly nuclear protein. Furthermore, we found a significant increase in HNRNPH1 expression in GBM tumor tissue compared to normal brain tissue (Fig. 1D and Supplementary Fig. 1), which was confirmed by subsequent analysis of the IHC staining using QuPath software (Fig. 1E). We next sought to investigate the contribution of tumor cells to *HNRNPH1* expression within the GBM tumor microenvironment through examination of a dataset of single-cell transcriptomes generated from 7930 cells across 28 GBM tumor samples, which included 25 newly diagnosed and three recurrent GBMs (GEO: GSE131928) [28] (Fig. 1F). By using the originally reported gene signatures, we confirmed that the cell groups formed distinct clusters by uniform manifold approximation and projection (UMAP) analysis (Fig. 1G). While *HNRNPH1* was expressed in all cell types (Fig. 1H), *HNRNPH1* median expression was highest in tumor cells (Fig. 1I).

### HNRNPH1 controls the expression of cell cycle-associated genes

**Fig. 2:**
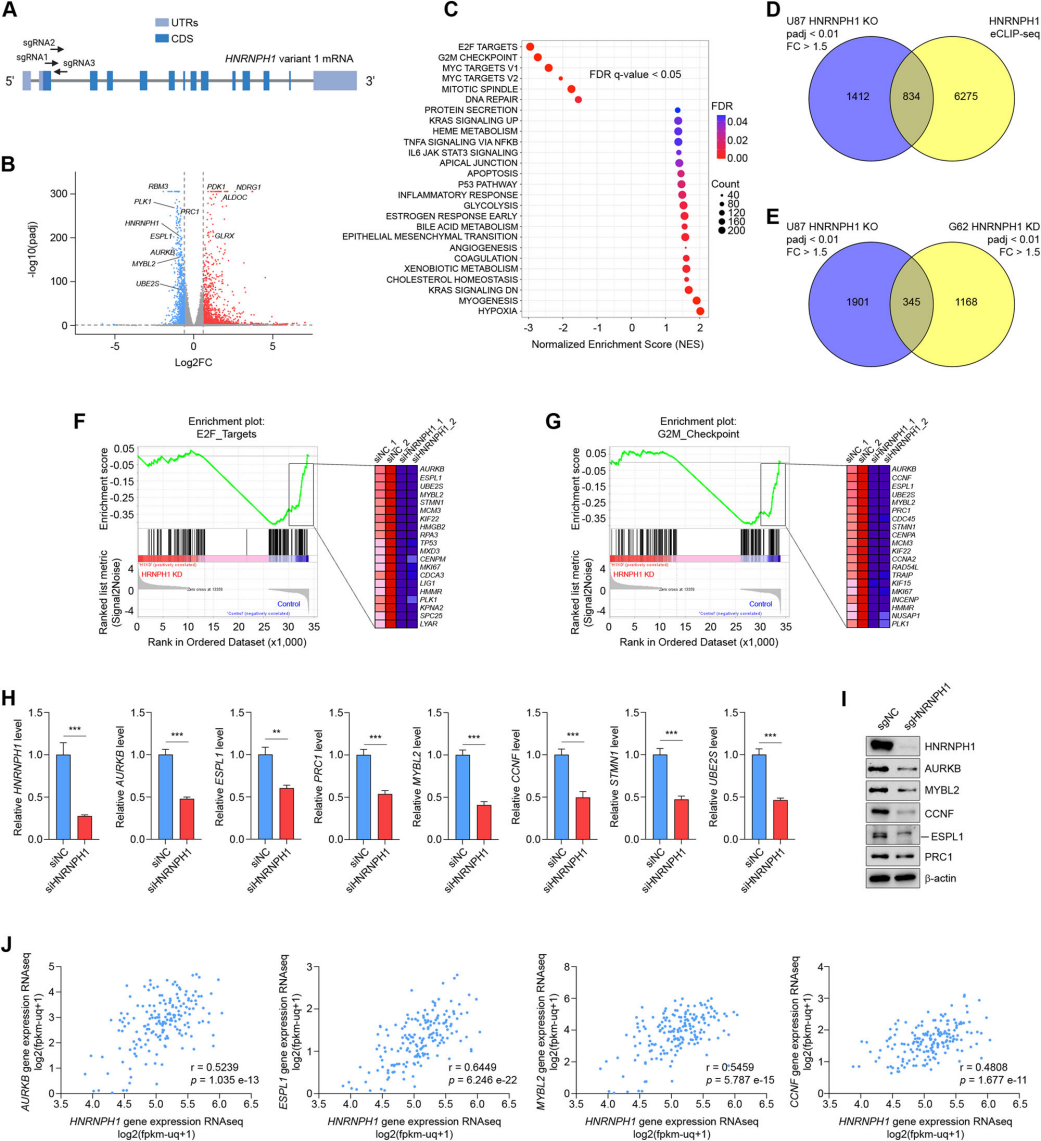
Knocking out HNRNPH1 reduces the expression of cell cycle-associated genes. **A** Schematic representation of the CRISPR/Cas9 strategy to knock out *HNRNPH1* in GBM cells. Three spatially coordinated synthetic single guide RNAs (sgRNAs) were designed to induce a fragment deletion between exon 2 and intron 2 of the coding sequence of *HNRNPH1* variant 1 (UTR = untranslated region; CDS = coding sequence). **B** Volcano plot comparing *HNRNPH1*-knockout U87 cells to control U87 cells. Genes colored in blue are significantly downregulated in the *HNRNPH1*-knockout cells, and genes colored in red are significantly upregulated in the *HNRNPH1*-knockout cells. **C** Dot plots of GSEA Hallmark analysis of enriched gene sets in *HNRNPH1*-knockout U87 cells compared to control U87 cells. The figure shows the significantly enriched gene sets at FDR q-value < 0.05. A positive Normalized Enrichment Score (NES) value indicates enrichment in the *HNRNPH1*-knockout cells, and a negative NES indicates enrichment in the control cells. Gene count refers to the number of genes associated with each gene set. **D** The Venn diagram shows the number of DEGs in *HNRNPH1*-knockout U87 cells (RNA-seq) relative to the number of genes identified to be directly bound by HNRNPH1 (eCLIP-seq). **E** The Venn diagram shows the number of DEGs in *HNRNPH1*-knockout U87 cells (RNA-seq, *n* = 3 biological replicates) compared to the number of DEGs in *HNRNPH1*-knockdown patient-derived G62 cells (RNA-seq, *n* = 2 biological replicates). **F**, **G** Enrichment plots for the two most enriched gene sets in control G62 cells from the GSEA Hallmark analysis. Shown are the enrichment plot for E2F targets (**F**, left panel) and the twenty most downregulated genes in *HNRNPH1*-knockdown (**F**, right panel); enrichment plot for G2/M checkpoint (**G**, left panel) and the twenty most downregulated genes in *HNRNPH1*-knockdown (**G**, right panel). **H** qRT-PCR analysis of *HNRNPH1*, *AURKB*, *ESPL1*, *PRC1*, *MYBL2*, *CCNF*, *STMN1*, and *UBE2S* expression in G62 cells transfected with a pool of control siRNAs or a pool of siRNAs targeting *HNRNPH1*. Data shown as mean ± SD of three replicates. Data were analyzed by unpaired t-test: **p < 0.01, ***p < 0.001. **I** Western Blot analysis of HNRNPH1, AURKB, MYBL2, CCNF, ESPL1, and PRC1 expression in G62 cells nucleofected with sgRNAs control or sgRNAs targeting *HNRNPH1*. **J** The correlation between *HNRNPH1* expression and the expression of *AURKB*, *ESPL1*, *MYBL2*, and *CCNF* in the TCGA GBM dataset.

To investigate the effects of HNRNPH1 silencing on global gene expression, we first knocked out *HNRNPH1* in U87 cells using a multi-guide strategy. Three spatially coordinated synthetic single guide RNAs (sgRNAs) were designed to induce a fragment deletion at the beginning of the coding sequence of *HNRNPH1* variant 1, which encodes the longest *HNRNPH1* isoform. Two sgRNAs targeted the second exon, and the third sgRNA targeted the proximal region of the second intron (Fig. 2A). After Cas9/sgRNAs transfection, the cells were kept as a pool, and reduced expression of HNRNPH1 was validated by immunoblot (Supplementary Fig. 2A). Silencing HNRNPH1 altered the expression of 2246 genes (padj < 0.01, fold change > 1.5, Fig. 2B and Supplementary Table 2). Gene set enrichment analysis (GSEA) of normalized counts of genes expressed in *HNRNPH1* knockout compared to control showed six gene sets significantly downregulated in cells with silenced *HNRNPH1* and 20 significantly enriched gene sets (FDR < 0.05). The downregulated gene sets included “E2F targets”, “G2/M checkpoint”, “MYC targets V1 and V2”, and “Mitotic spindle” (Fig. 2C). These gene sets encompassed genes related to cell proliferation and cell division. The other downregulated gene set was “DNA repair”. The enriched gene sets included “hypoxia”, “angiogenesis”, “inflammatory response”, “TNFA signaling via NFkB”, and “protein secretion”. Notably, analysis of the enhanced CLIP sequencing (eCLIP-seq) data we have previously generated (GEO: GSE137489) [26] showed that of the 2246 differentially expressed genes (DEGs), 834 were found to be directly bound by HNRNPH1 (Fig. 2D). To validate HNRNPH1 effects on gene expression in PDGCs and identify the genes that are deregulated after a short-term silencing of HNRNPH1, we transfected patient-derived G62 cells with a pool of control siRNAs or siRNAs targeting *HNRNPH1* (Supplementary Fig. 2B). We found 1513 genes differentially expressed in G62 with silenced *HNRNPH1* (padj < 0.01, fold change > 1.5, Supplementary Fig. 2C and Supplementary Table 3), of which 345 genes were commonly deregulated in U87 and G62 (Fig. 2E). GSEA of normalized counts of genes expressed in G62 HNRNPH1 knockdown compared to control confirmed that “E2F targets” and “G2/M checkpoint” were the two most significantly downregulated gene sets (Fig. 2F, G and Supplementary Fig. 2D). Therefore, we further validated the expression of the most significantly deregulated cell cycle genes that were directly bound by HNRNPH1 using qPCR in G62 PDGCs. Our data confirmed that silencing *HNRNPH1* reduced mRNA levels of *AURKB*, *ESPL1*, *PRC1*, *UBE2S*, *MYBL2*, *CCNF*, and *STMN1* (Fig. 2H). Exogenous expression of HNRNPH1 in knockout U87 cells was sufficient to restore the expression of all the cell cycle-associated genes analyzed except *STMN1* (Supplementary Fig. 2F). Moreover, knocking out HNRNPH1 in G62 resulted in the downregulation of the protein levels of AURKB, MYBL2, CCNF, ESPL1, and PRC1 (Fig. 2I), but not UBE2S and STMN1 (Supplementary Fig. 2E). Notably, TCGA data analysis of 175 GBMs revealed that *HNRNPH1* expression was significantly correlated with *AURKB* (*r* = 0.5239, *p* = 1.035e-13), *ESPL1* (*r* = 0.6449, *p* = 6.246e-22), *MYBL2* (*r* = 0.5459, *p* = 5.787e-15), *CCNF* (*r* = 0.4808, *p* = 1.677e-11), and *PRC1* levels (*r* = 0.4388, *p* = 1.256e-9) (Fig. 2J and Supplementary Fig. 2G). We also observed a significant but weak correlation with *UBE2S* (*r* = 0.3453, *p* = 2.878e-6) and no correlation with *STMN1* (*r* = 0.0353, *p* = 0.6428) (Supplementary Fig. 2G).

### HNRNPH1 expression pattern is associated with proliferation-related gene abundance

**Fig. 3:**
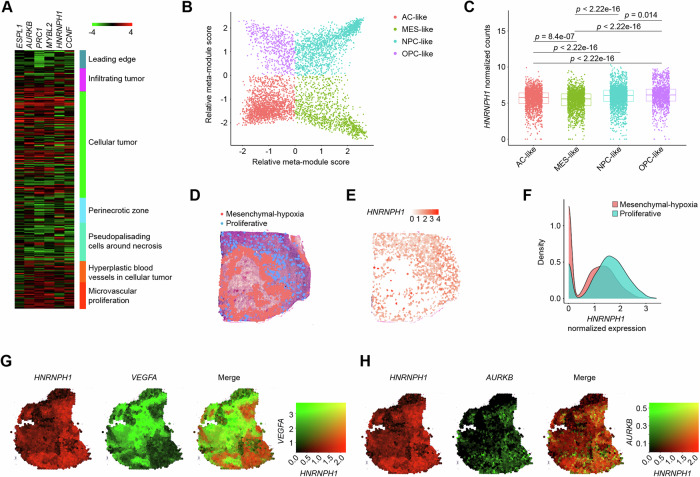
Spatial distribution of *HNRNPH1* expression. **A** Heatmap of the expression levels (RNA-seq) of *HNRNPH1* and cell cycle-associated genes in different anatomical regions of GBM. **B** Two-dimensional visualization of cellular states. Each color corresponds to one cellular state: AC-like (red), MES-like (green), NPC-like (light blue), and OPC-like (purple). The position of the dots reflects their relative scores for the meta-modules. **C** Box plot of *HNRNPH1* expression (normalized counts) between AC-like (red), MES-like (green), NPC-like (light blue), and OPC-like (purple). Data were analyzed by the Wilcoxon test (n = 6863). **D** Spatial map of sample UKF313 showing spot annotation by MP. Shown are the mesenchymal-hypoxia (red) and the proliferative (blue) MPs. **E** Spatial map of *HNRNPH1* expression in sample UKF313. **F** Density plots of *HNRNPH1* expression in mesenchymal-hypoxia (red) and proliferative (blue) MPs using normalized read count data (*n* = 10767 spots across 26 GBM sections). **G**, **H** Spatial map of *HNRNPH1* expression (red) and *VEGFA* expression (green, **G**) or *AURKB* expression (green, **H**) in sample UKF255.

As noted above, *HNRNPH1* exhibited higher expression in GBM tumor cells than in stromal cells (Fig. 1I). Since GBM cancer cells have been shown to exist in different cellular states that organize to form local environments [29], we assessed whether *HNRNPH1* expression is associated with a particular cell state or spatial localization. First, we analyzed RNA sequencing data of 122 RNA samples from ten GBM tumors divided into seven anatomic structures (leading edge, infiltrating tumor, cellular tumor, perinecrotic zone, pseudopalisading cells around necrosis, hyperplastic blood vessels in cellular tumor, and microvascular proliferation). *HNRNPH1* and *HNRNPH1*-regulated cell cycle genes were mainly found in the cellular tumor, infiltrating tumor, and microvascular areas but not in the necrotic areas (Fig. 3A), which we previously showed to express a hypoxic signature [27]. We next analyzed *HNRNPH1* expression in the single-cell RNAseq dataset (GEO: GSE131928) [28]. We classified the cell states from all tumors as neural-progenitor-like (NPC-like), oligodendrocyte-progenitor-like (OPC-like), astrocyte-like (AC-like), and mesenchymal-like (MES-like) by using the original meta-module assignments (Fig. 3B). We found that *HNRNPH1* was significantly more expressed in the NPC- and OPC-like states (Fig. 3C), which were shown to express a cell cycle signature [28]. On the contrary, the MES-like state, which was associated with a hypoxic signature, had the lowest *HNRNPH1* median expression (Fig. 3C). To spatially localize *HNRNPH1* expression, we analyzed the spatial transcriptomic profile of 26 GBM sections from an external dataset (GEO: GSE237183) [29]. While 14 GBM spatial metaprograms (MPs) were originally identified, due to the transcriptomic effects of *HNRNPH1* knockout, we focused on the mesenchymal-hypoxia (MES-hypoxia) and proliferative MPs (Fig. 3D). We found that the proliferative MP had higher expression of *HNRNPH1* than the MES-hypoxia MP (Fig. 3E, F). Moreover, *HNRNPH1* had higher regional co-expression with *AURKB*, *ESPL1*, *MYBL2*, *CCNF*, and *PRC1* than with *VEGFA*, used as a marker of hypoxic areas (Fig. 3G, H and Supplementary Fig. 3A–D).

### HNRNPH1 regulates the alternative splicing of cell cycle-associated genes

**Fig. 4:**
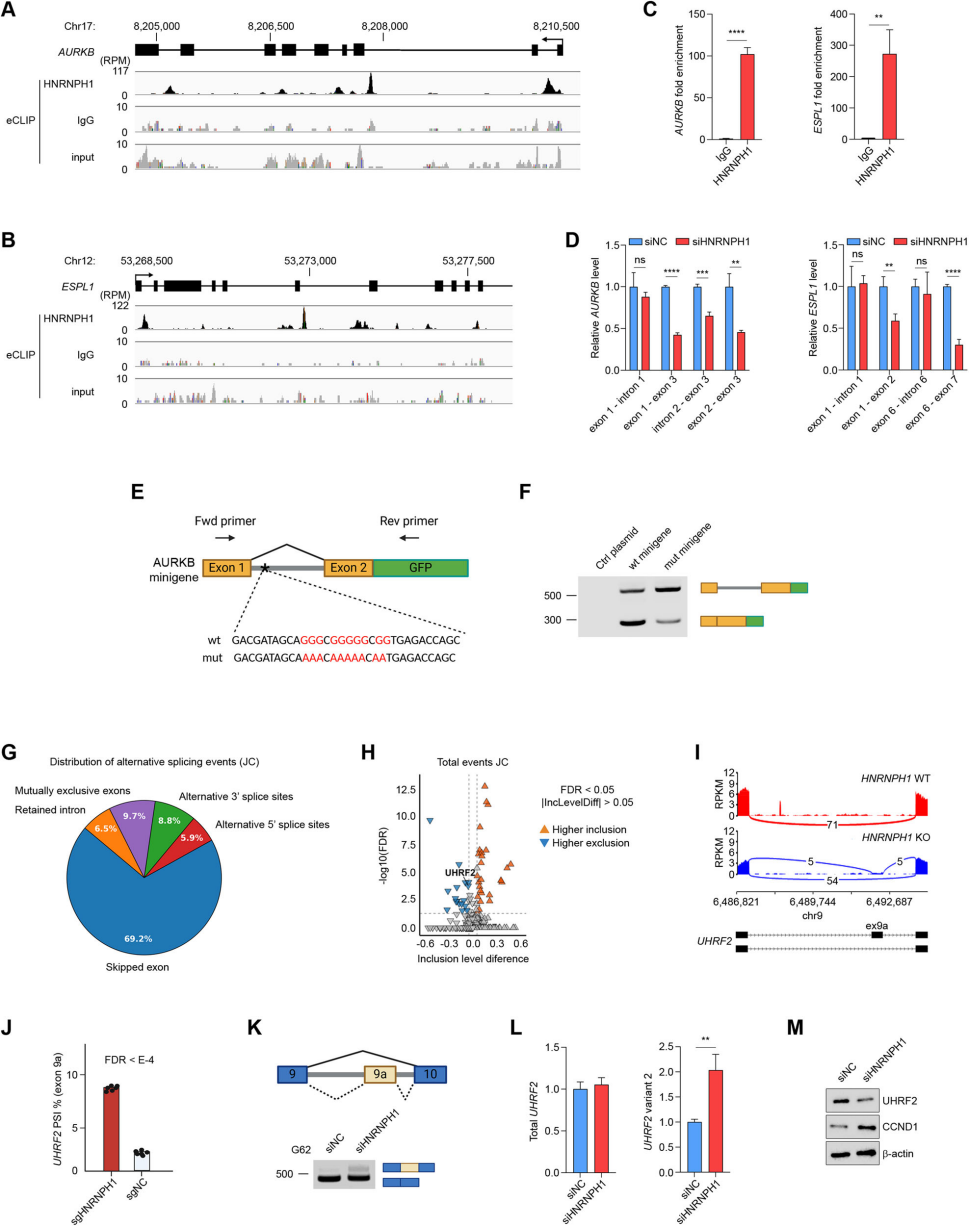
HNRNPH1 regulates the alternative splicing of cell cycle-associated genes. **A**, **B** Analysis of HNRNPH1 binding sites in the *AURKB* (**A**) and *ESPL1* (**B**) pre-mRNA by eCLIP. Read density in reads per million (RPM) is shown for HNRNPH1, IgG, and input. **C** HNRNPH1 RIP (RNA immunoprecipitation) followed by qRT-PCR analysis of co-purified *AURKB* (left) and *ESPL1* (right) in UV-crosslinked PDGCs. **D** qRT-PCR analysis of exon-intron and exon-exon junction for *AURKB* (left) and *ESPL1* (right). **E** Diagram of AURKB minigene splicing with primers used for PCR analysis (top). Wild-type (wt) and mutant (mut) sequences in the intronic region of the minigene are shown (bottom). Red letters represent the mutated nucleotides. **F** Splicing pattern of the transfected empty control plasmid, wt AURKB minigene, and mut AURKB minigene in HEK293 cells. **G** Distribution of alternative splicing events detected using the Junction Count (JC) approach. **H** Differentially spliced events detected by the JC approach. Inclusion level differences (U87 Control vs. *HNRNPH1*-knockout) on the x-axis and statistical significance (-log10 FDR) on the y-axis. Significant events (FDR < 0.05, |ΔPSI | > 0.05) are highlighted for higher inclusion (orange) and higher exclusion (blue). **I** Sashimi plot showing the inclusion of *UHRF2* exon 9a in U87 *HNRNPH1*-knockout (blue) compared to U87 *HNRNPH1* wild-type (red). **J** Analysis of *UHRF2* exon 9a percent inclusion (PSI%) in U87 *HNRNPH1*-knockout compared to U87 control. **K** Diagram of *UHRF2* AS (top) and PCR analysis of *UHRF2* splicing pattern in G62 PDGCs transfected with siRNA control or siRNA targeting *HNRNPH1* (bottom). **L** qRT-PCR analysis of total *UHRF2* (left) and *UHRF2* variant 2 (exon 9-exon 9a junction) expression in G62 PDGCs transfected with siRNA control or siRNA targeting *HNRNPH1*. **M** Western Blot analysis of UHRF2 and CCND1 expression in G62 PDGCs transfected with siRNA control or siRNA targeting *HNRNPH1*. Data shown as mean ± SD of three replicates. Data were analyzed by unpaired t-test: **p < 0.01, ***p < 0.001, ****p < 0.0001.

To identify the mechanisms through which HNRNPH1 regulates cell cycle-associated gene expression, we first used our eCLIP-sequencing data (GEO: GSE137489) [26] to map HNRNPH1 binding sites on the pre-mRNAs of *AURKB* and *ESPL1*. We identified two major peaks on the proximal intron 1 and distal intron 2 of *AURKB* (Fig. 4A), and two major peaks on the proximal intron 1 and proximal intron 6 of *ESPL1* (Fig. 4B). We validated HNRNPH1 interaction with *AURKB* and *ESPL1* by in vivo RNA UV crosslinking and immunoprecipitation (Fig. 4C). By designing primers targeting the exon-intron junctions or the exon-exon junctions, we found that silencing HNRNPH1 reduced the expression of spliced *AURKB* and *ESPL1* with no significant changes in the unspliced RNAs (Fig. 4D). To further validate HNRNPH1’s role in cell cycle gene splicing, we generated a minigene construct containing the 399 nt genomic sequence spanning *AURKB* exon1-2, with the entire intron 1, upstream of a GFP gene (Fig. 4E). Then, we generated a mutant version of the minigene by substituting the G-stretch sequence of the major eCLIP-seq peak found in intron 1 with an A-stretch (Fig. 4E). Transient expression of the two minigenes showed significant intron retention in the mutant minigene compared to the wt minigene (Fig. 4F). A GFP plasmid lacking the minigene was used as a control. Together, these data suggest that HNRNPH1 binds *AURKB* and *ESPL1* to regulate their splicing.

To investigate HNRNPH1 regulation of AS events, we analyzed our RNA-seq data from *HNRNPH1*-knockout U87 cells using the rMATS software to generate splice JC and splice junction exon counts (JCEC). Among the different classes of AS analyzed (retained intron, skipped exon, alternative 5’/3’ splice site, and mutually exclusive exons), we identified skipped exons as the predominant event in both JC and JCEC (Fig. 4G, Supplementary Fig. 4A, B, and Supplementary Table 4). We then analyzed AS events of cell cycle-associated genes and observed a significant splicing alteration of the *UHRF2* gene in both JC and JCEC (Fig. 4H and Supplementary Fig. 4C). UHRF2 is a ubiquitin ligase that occupies a central position in the cell cycle network by coordinating several components of the cell cycle machinery [30]. The inclusion of an alternative internal exon in the coding region of *UHRF2* results in a frameshift and the introduction of a premature stop codon, rendering the *UHRF2* transcript a potential target for nonsense-mediated RNA decay (NMD). Differential splicing analysis showed that *UHRF2* exon 9a inclusion levels increased upon *HNRNPH1* silencing (Fig. 4I, J). The inclusion of exon 9a was associated with a significant reduction in *UHRF2* levels in *HNRNPH1*-knockout cells (Supplementary Fig. 4D). We validated exon 9a inclusion in G62 PDGCs transfected with siRNAs targeting *HNRNPH1* (Fig. 4K). While transient *HNRNPH1* silencing in G62 did not change the levels of total *UHRF2* mRNA, we found a two-fold increase in *UHRF2* variant 2, which presents exon 9a inclusion (Fig. 4L). Increased inclusion of exon 9a was associated with reduced UHRF2 protein levels and upregulation of cyclin D1, a well-known target of UHRF2 (Fig. 4M). Interestingly, the eCLIP-sequencing identified binding of HNRNPH1 to intron 12 and the 3’ UTR region of *UHRF2* but not exon 9a (Supplementary Fig. 4E). Therefore, we first validated the binding of HNRNPH1 to the *UHRF2* mRNA by in vivo RNA UV crosslinking and immunoprecipitation experiments using G62 PDGCs (Supplementary Fig. 4F). Next, to investigate the effects of HNRNPH1 binding to intron 12 on *UHRF2* AS, we used a CRISPR/Cas9 system to induce a fragment deletion of the HNRNPH1 binding site (Supplementary Fig. 4G). We confirmed deletion of the HNRNPH1 binding site in intron 12 by PCR (Supplementary Fig. 4H). Deletion of the HNRNPH1 binding site resulted in the reduction of exon 9-exon 10 junction in the *UHRF2* mRNA, with no change in the level of exon 9-exon 9a junction (Supplementary Fig. 4I). Together, these findings suggest a critical role of HNRNPH1 in *UHRF2* AS.

### Silencing HNRNPH1 leads to reduced proliferation and cell cycle arrest

**Fig. 5:**
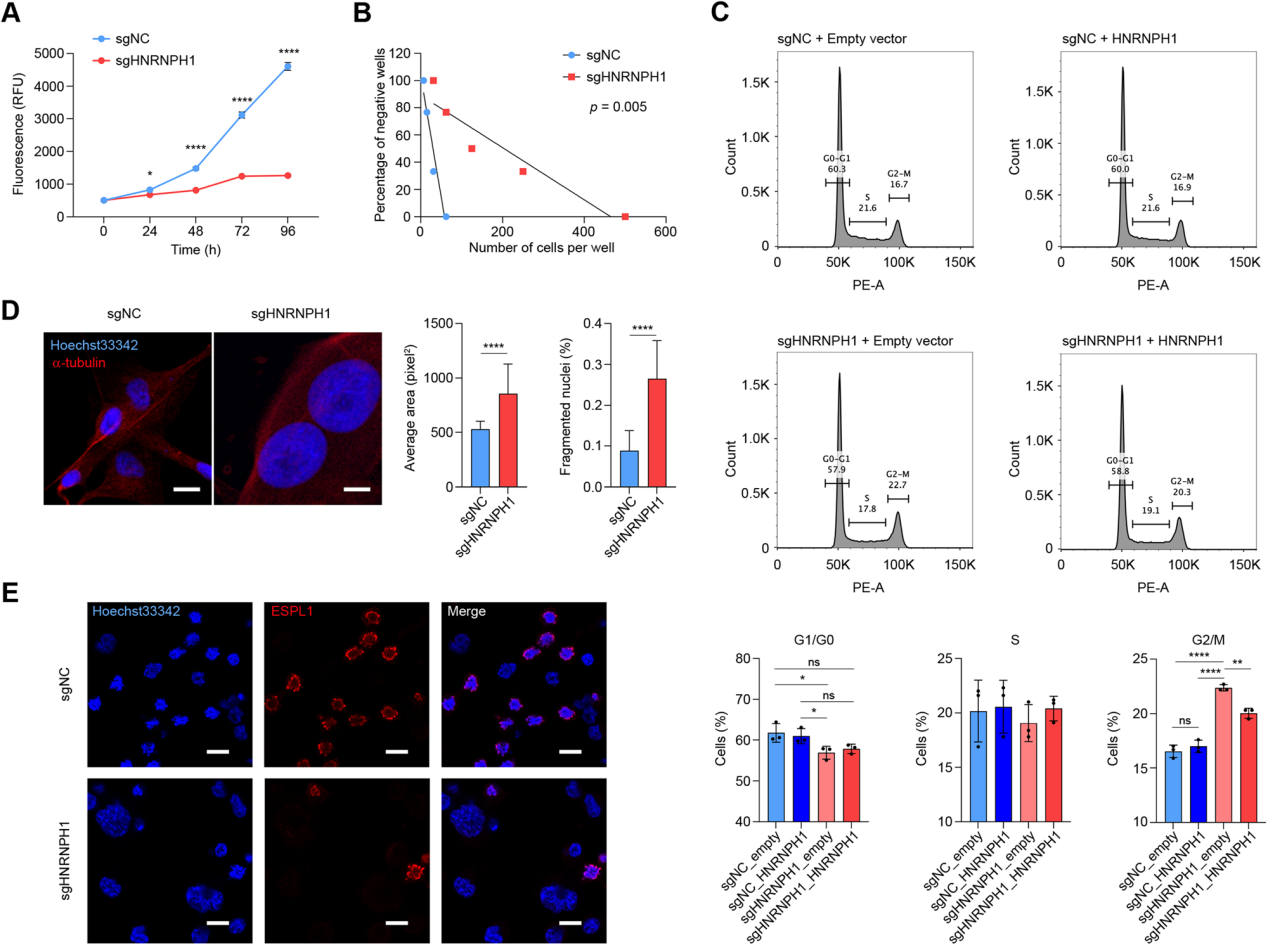
Silencing HNRNPH1 reduces GBM cell proliferation. **A** Cell viability assay of G62 PDGCs nucleofected with sgRNA control or sgRNAs targeting *HNRNPH1*. Data shown as mean ± SD of three replicates. Data were analyzed by two-way ANOVA: *p < 0.05, ****p < 0.0001. **B** Analysis of sphere formation capacity by limiting dilution assay of G62 PDGCs nucleofected with sgRNA control or sgRNAs targeting *HNRNPH1*. The amount of initially seeded cells (x-axis) is plotted against the percentage of wells without any detected spheres (y-axis). **C** Flow cytometry analysis for cell cycle distribution of G62 cells nucleofected with sgRNAs control or sgRNAs targeting *HNRNPH1*, followed by nucleofection with an empty vector or human HNRNPH1. Bar graphs show the quantification of cells in G1 (left), S (center), and G2/M (right). Data shown as mean ± SD of three replicates. Data were analyzed by unpaired t-test: *p < 0.05, **p < 0.01, ****p < 0.0001. **D** Representative microphotographs of U87 control (left) and *HNRNPH1*-knockout (right) cells stained for alpha-tubulin (red) and nuclei (Hoechst 33342). The bar graphs show the relative nuclear size (left) and the percentage of fragmented nuclei (right). Scale bar 10 µm. ****p < 0.0001. **E** Representative microphotographs of G62 control (top) and *HNRNPH1*-knockout (bottom) cells stained for separase (ESPL1, red) and nuclei (Hoechst 33342, blue). Cells were synchronized in the G2/M phase by incubation in nocodazole. Scale bar 10 µm.

To dissect the effects of HNRNPH1 on GBM cells, we first knocked out *HNRNPH1* in the patient-derived G62 cells by nucleofecting them with the sgRNAs targeting *HNRNPH1*. Compared to the cells nucleofected with an sgRNA control, *HNRNPH1*-knockout cells showed significantly slower proliferation and complete growth arrest between 72 and 96 h (Fig. 5A). Since HNRNPF, a close homolog of HNRNPH1, was also found upregulated in GBM, we next analyzed the effect of HNRNPF silencing on the growth of wild-type and HNRNPH1-knockout cells. We found that targeting HNRNPF had a small but significant reduction in the growth of HNRNPH1 wild-type cells, while a more dramatic effect on PDGC proliferation occurred when HNRNPH1 knockout was combined with HNRNPF silencing (Supplementary Fig. 5A, B). Moreover, silencing *HNRNPH1* led to diminished neurosphere-forming capacity of PDGCs and smaller neurosphere size (Fig. 5B and Supplementary Fig. 5C). To examine whether the reduced cell proliferation is due to alterations in the cell cycle, we labeled U87 and G62 cells with propidium iodide and performed FACS analysis. We found that both *HNRNPH1*-knockout U87 and G62 cells exhibited an increased number of cells arrested in the G2/M phase, which was associated with a reduction in the percentage of cells in the G1 phase. No significant changes were observed in the S phase (Fig. 5C and Supplementary Fig. 5D). Exogenous expression of HNRNPH1 in knockout cells rescued the mitotic phenotype, resulting in a substantial reduction in the number of cells arrested in the G2/M phase (Fig. 5C). These results suggested that silencing HNRNPH1 may interfere with the cell’s ability to divide. Therefore, we next stained nuclei with Hoechst 33342 to analyze their morphology. We found that the nuclei of *HNRNPH1*-knockout cells were significantly larger than control cells (Fig. 5D). Moreover, *HNRNPH1*-knockout cells showed a significant increase in the number of polynucleated cells and cells with fragmented nuclei (Fig. 5D). Since we found that HNRNPH1 controls the expression of AURKB (Aurora B kinase) and ESPL1 (Separase), two proteins important for sister chromatid separation during mitosis [31], we examined the subcellular distribution of Aurora B and Separase in G62 PDGCs after knocking out *HNRNPH1*. To increase the number of cells in the G2/M phase, we treated them with nocodazole. As shown in Fig. 5E and Supplementary Fig. 5E, Aurora B and Separase were detected only in cells undergoing mitosis. Moreover, Aurora B and Separase were found associated with chromosomes condensed and chromosomes aligned at the center of the mitotic spindle (Fig. 5E and Supplementary Fig. 5E, F). *HNRNPH1*-knockout cells presented fewer Aurora B- and Separase-positive cells undergoing mitosis and an increased number of cells with large nuclei (Fig. 5E and Supplementary Fig. 5E).

### HNRNPH1 promotes tumor growth

**Fig. 6:**
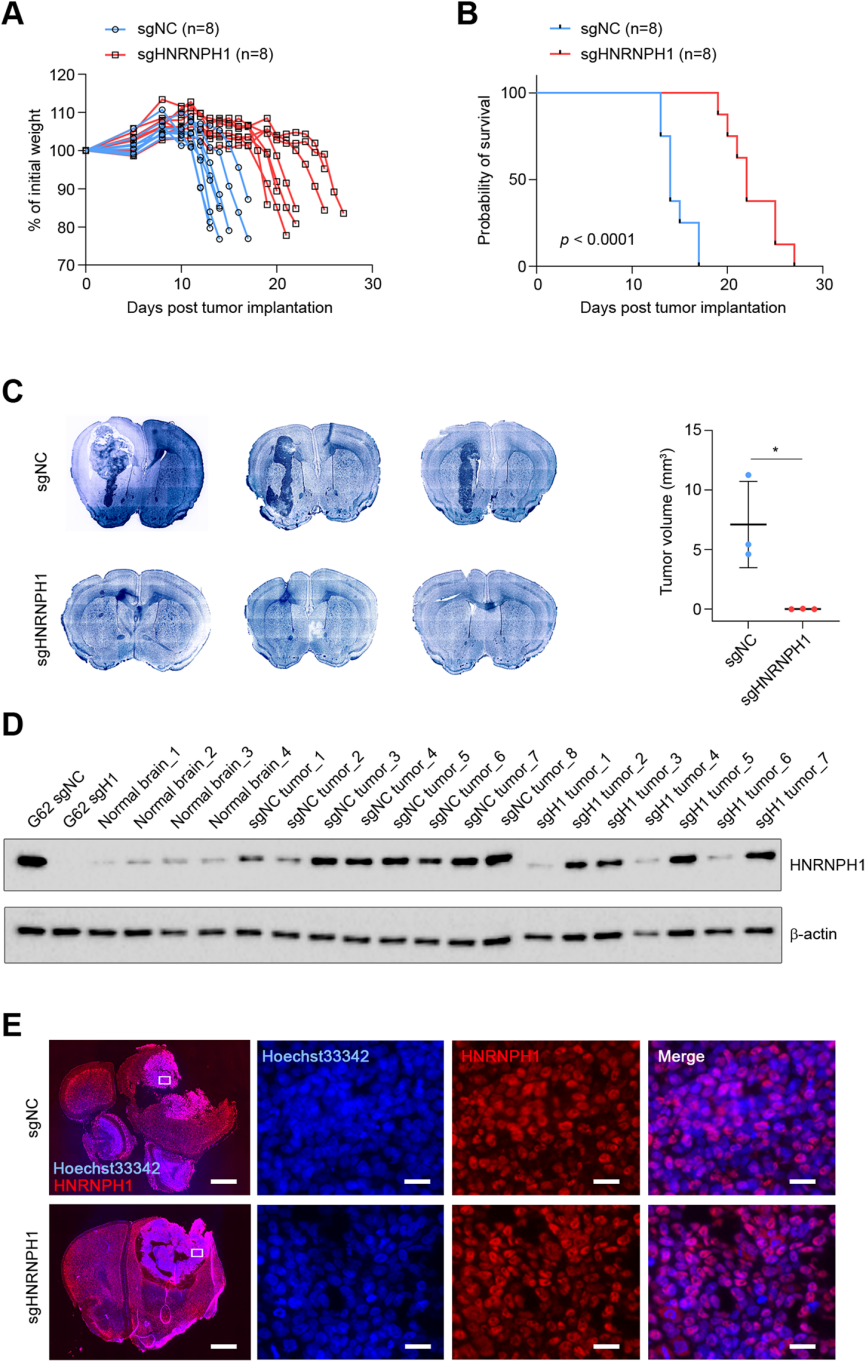
Silencing HNRNPH1 reduces tumor progression in vivo. **A** Body weight changes (percentage over the weight at the time of tumor implantation) in mice injected with control patient-derived G62 cells (*n* = 8) and *HNRNPH1*-knockout G62 cells (*n* = 8). **B** Kaplan-Meier survival curves of mice injected with control G62 cells (*n* = 8) and *HNRNPH1*-knockout G62 cells (*n* = 8). **C** Hoechst 33342 staining of brain sections (left) and tumor volume quantification (right) in control (*n* = 3) and *HNRNPH1*-knockout (*n* = 3) mice 11 days post-tumor implantation. Data shown as mean ± SD. Data were analyzed by unpaired t-test: *p < 0.05. **D** Western Blot analysis of HNRNPH1 expression in G62 cells at the time of implantation, mouse normal brain, and terminal control and *HNRNPH1*-knockout tumors. **E** Immunofluorescence of brain sections with terminal control (top) and HNRNPH1-knockout (bottom) tumors stained for HNRNPH1 (red) and nuclei (Hoechst 33342, blue). The white square represents the magnified area. Scale bar in whole brain microphotographs 1000 µm. The scale bar in the magnified images is 25 µm.

To validate the impact of HNRNPH1 on PDGC tumorigenicity in vivo, we first attempted to generate *HNRNPH1*-knockout clones from the G62 PDGCs. Of the 110 clones plated, we observed the growth of only two clones. However, these two clones exhibited HNRNPH1 protein levels comparable to those of the control cells (Supplementary Fig. 6A), suggesting that HNRNPH1 was not successfully knocked out in those cells. Therefore, we intracranially injected control and HNRNPH1-knockout patient-derived G62 cells into mice as a pool with no clonal selection. We observed that all the injected mice gained weight in the first 10 days post-surgery. Mice in the control group started losing weight on day 12, and all the mice lost more than 15% of their body weight by day 17. On the contrary, the mice in the *HNRNPH1*-knockout group remained stable for a longer period and showed a significant amount of body weight loss between days 19 and 27 (Fig. 6A). This was associated with significant survival benefits in the *HNRNPH1*-knockout group (Fig. 6B). To carry out tumor volume analysis, we sacrificed one group of mice on day 11 post-injection and performed brain sectioning. While strikingly large tumors were observed in the control group, only one out of three mice of the *HNRNPH1*-knockout group showed a small measurable tumor (Fig. 6C). Since all the mice in the knockout group died by day 27, we hypothesized that tumor formation was promoted by cells in the pool that did not successfully knockout *HNRNPH1*, which ultimately resulted in tumor progression and only modest survival benefits. To test our hypothesis, we analyzed the expression of HNRNPH1 in mouse tumors that reached the endpoint by immunoblot. Lysates from four normal mouse brains were used as a control for basal HNRNPH1 expression. Of note, one of the eight mice implanted with *HNRNPH1*-knockout cells did not show evidence of tumor formation even if symptomatic (Supplementary Fig. 6B). As expected, the expression of HNRNPH1 was significantly reduced in the knockout cells before implantation. On the contrary, in terminal knockout tumors, the expression of HNRNPH1 reached levels that were comparable to those in control tumors (Fig. 6D). Expression of HNRNPH1 in the tumors was validated by immunostaining of brain sections at both 11 days and endpoint (Fig. 6E and Supplementary Fig. 6C). However, three of the seven tumors analyzed by immune blot did not show HNRNPH1 upregulation. Therefore, we extracted the RNA from knockout tumors of mice 1 and 4 to validate the presence of deletions or mutations in the *HNRNPH1* sequence. No tumor specimen was available for RNA extraction from mouse 6. While a wild-type HNRNPH1 sequence was found in mouse 4 (Supplementary Fig. 6D), an in-frame deletion in the *HNRNPH1* sequence was present in mouse 1 (Supplementary Fig. 6E), which deleted three amino acids at the N-terminus of HNRNPH1 without altering the rest of the protein (Supplementary Fig. 6F). Together, these results support our original hypothesis that tumor growth in the knockout mice was due to the proliferation of clones with intact HNRNPH1 expression.

## Discussion

The most fundamental trait of cancer cells is their ability to sustain chronic proliferation [32]. A better understanding of the molecular mechanisms that control cell cycle progression in highly aggressive and deadly tumors, such as GBM, is paramount to developing alternative and more effective therapies. Our study identifies the RNA-binding protein HNRNPH1 as a critical regulator of GBM proliferation and mitotic integrity. We demonstrate that HNRNPH1 is overexpressed in GBM, where it plays a crucial role in maintaining the expression and proper splicing of genes essential for cell cycle progression. Together, these findings suggest that HNRNPH1 serves as a key safeguard against replication stress, thereby promoting tumor progression.

Continuous proliferation of cancer cells is closely linked to the activation of the E2F-dependent transcriptional network [33]. GBM, as well as many other cancers, harbors mutations that induce E2F-dependent transcription, promoting cell cycle entry and increased cell division rates [2–5]. Notably, genes of the replication stress checkpoint and spindle assembly are rarely mutated, and they are essential to prevent catastrophic levels of DNA damage that may impact cell viability [6]. Using loss-of-function experiments, we demonstrated that HNRNPH1 is a key regulator of many genes of the G2/M checkpoint and E2F targets, which orchestrate proper chromosome segregation and mitosis. Several of these genes, including *AURKB* and *ESPL1*, were directly bound by HNRNPH1 in intronic regions. Interestingly, only introns bound by HNRNPH1 in the proximal region/5’ splicing site were retained upon HNRNPH1 silencing. This finding aligns with previous studies showing that the specific binding site of RBPs on a pre-mRNA defines their splicing function [34]. However, validation on a larger number of genes will be essential to determine if this association between the HNRNPH1 binding site and the type of splicing event applies to all the targets.

In addition to splicing-activating functions, the binding of HNRNPH1 to G-rich RNA sequences plays a crucial role in regulating AS [35]. Here, we identified a novel AS event regulated by HNRNPH1. The binding of HNRNPH1 to intron 12 of the cell cycle-associated gene *UHRF2* mediates exon 9a skipping and expression of the coding mRNA isoform. Silencing HNRNPH1 promotes the inclusion of exon 9a, resulting in the production of a noncoding RNA variant and the downregulation of UHRF2 protein. UHRF2 has been mainly studied for its role in the G1 phase of the cell cycle [36]. However, its ability to interact with several different cyclins and cyclin-dependent kinases (CDKs) suggests a broader role of this ubiquitin ligase in cell cycle progression [30]. UHRF2 can function as a tumor suppressor or oncogene depending on the type of cancer [37, 38], but there is evidence suggesting that UHRF2 is highly expressed in the brain [39] and its silencing inhibits the growth of U251 GBM cells [40]. Therefore, AS of *UHRF2* may contribute to the HNRNPH1-dependent regulation of cell proliferation.

In comparison to prior studies on RNA splicing in GBM [41], our results highlight a central role for HNRNPH1 in the post-transcriptional regulation of a multitude of cell cycle-associated network modules, thereby promoting tumor progression. Loss of HNRNPH1 led to abnormal nuclear morphology and cell cycle arrest. Moreover, we demonstrated that HNRNPH1 expression was essential for tumor development in vivo. To the best of our knowledge, our work is also the first to show higher HNRNPH1 expression levels in specific tumor cell states and locations. Our data suggest that hypoxia, a major driver of tissue structure and spatial organization [29], is a key factor in shaping HNRNPH1 expression patterns. Hypoxia is linked to the mesenchymal cell state [28], which showed the lowest HNRNPH1 levels. This finding is supported by the RNA-sequencing analysis of GBM cells in which silencing of HNRNPH1 led to the upregulation of hypoxia-associated genes. However, GBM is a very heterogeneous tumor, and each tumor is composed of multiple cellular states. These cellular states are dynamic, and each cell can proliferate and transition to another state [28]. Therefore, we propose that HNRNPH1 plays a critical role in tumor progression, independent of the cellular state.

Therapeutically, targeting HNRNPH1 may represent a novel strategy to block cell cycle progression in GBM cells. Given the growing interest in RNA-targeted therapies, future work should explore the development of small-molecule inhibitors or antisense oligonucleotides designed to disrupt HNRNPH1 functions. Additionally, our findings suggest that combining HNRNPH1 inhibition with existing mitotic checkpoint-targeting drugs could enhance therapeutic efficacy by exacerbating mitotic failure in GBM cells.

Despite these promising insights, several questions remain. Future studies should investigate whether HNRNPH1 exerts similar roles in other cancers and whether its inhibition selectively targets tumor cells while sparing normal tissues, including those in the central nervous system. One limitation of our study is the depth of our RNA-sequencing analysis, which may have significantly reduced the number of AS events detected. Therefore, further characterization of the AS events regulated by HNRNPH1 will provide deeper mechanistic insight into its oncogenic function.

In conclusion, our study establishes HNRNPH1 as an essential player in the post-transcriptional control of cell cycle genes that contribute to GBM progression. By elucidating the role of HNRNPH1 in sustaining mitotic integrity, we provide a new framework for understanding GBM proliferation and identify RNA splicing as a promising avenue for therapeutic intervention.

## Supplementary information

Supplementary Figure and Table legends

Supplementary Figure 1

Supplementary Figure 2

Supplementary Figure 3

Supplementary Figure 4

Supplementary Figure 5

Supplementary Figure 6

Supplementary Table 1

Supplementary Table 2

Supplementary Table 3

Supplementary Table 4

Original western blots

## Data Availability

The datasets generated and/or analyzed during the current study are available in the Gene Expression Omnibus (GEO), GSE294242, GSE294345, and GSE137489.
