# Supplementary material for: HNRNPH1 drives glioblastoma progression by regulating the splicing of cell cycle genes
Source: Cell Death Dis. 2026 Mar 24;17(1):352. doi: 10.1038/s41419-026-08576-6 (PMC13039110; doi:10.1038/s41419-026-08576-6)
Supplement: Supplementary file 1 — Supplementary Figure and Table legends [file 41419_2026_8576_MOESM1_ESM.docx]

**Supplementary Figure and Table Legends**

**Supplementary Fig. 1:** **HNRNPH1 expression levels are elevated in GBM tissue array.** Immunohistochemical staining of HNRNPH1 in GBM (A-G) and normal brain (H) tissues from a tissue microarray.

**Supplementary Fig. 2: HNRNPH1 regulates the expression of cell cycle-associated genes. A** Western Blot analysis of HRNRPH1 expression in U87 cells transfected with sgRNAs control or sgRNAs targeting *HNRNPH1*. **B** qRT-PCR analysis of HNRNPH1 expression in G62 cells transfected with a pool of siRNAs control or a pool of siRNAs targeting *HNRNPH1*. Data shown as mean ± SD of three replicates. Data were analyzed by unpaired t-test: ****p < 0.0001. **C** Volcano plot comparing *HNRNPH1*-knockout G62 cells to control G62 cells. Genes colored in blue are significantly downregulated in the *HNRNPH1*-knockout cells, and genes colored in red are significantly upregulated in the *HNRNPH1*-knockout cells. **D** GSEA Hallmark analysis of enriched gene sets in *HNRNPH1*-knockout G62 cells compared to control G62 cells. A positive Normalized Enrichment Score (NES) value indicates enrichment in the *HNRNPH1*-knockout cells, and a negative NES indicates enrichment in the control cells. **E** Western Blot analysis of UBE2S and STMN1 expression in G62 cells nucleofected with sgRNAs control or sgRNAs targeting *HNRNPH1*. **F** qRT-PCR analysis of *AURKB*, *ESPL1*, *PRC1*, *MYBL2*, *CCNF*, *STMN1*, and *UBE2S* expression in U87 cells transfected with control sgRNAs or sgRNAs targeting HNRNPH1 with or without exogenous HNRNPH1 expression. Data shown as mean ± SD of three replicates. Data were analyzed by unpaired t-test: ***p < 0.001, ****p < 0.0001. **G** The correlation between *HNRNPH1* expression and the expression of *PRC1*, *UBE2S*, and *STMN1* in the TCGA GBM dataset.

**Supplementary Fig. 3: Spatial co-expression analysis of HNRNPH1 and cell cycle-associated genes in GBM.** **A-D** Spatial map of *HNRNPH1* expression (red) and *ESPL1* (green, **A**), *PRC1* (green, **B**), *MYBL2* (green, **C**), and *CCNF* expression (green, **D**) in sample UKF255.

**Supplementary Fig. 4: HNRNPH1 regulates AS of *UHRF2*. A** Number of alternative splicing events detected using Junction Count (JC) and Junction Count Exon Count (JCEC) approaches. **B** Distribution of alternative splicing events detected using the Junction Count (JCEC) approach. **C** Differentially spliced events detected by the JCEC approach. Inclusion level differences (U87 Control vs. *HNRNPH1*-knockout) on the x-axis and statistical significance (-log10 FDR) on the y-axis. Significant events (FDR < 0.05, |ΔPSI| > 0.05) are highlighted for higher inclusion (orange) and higher exclusion (blue). **D** *UHRF2* expression (normalized counts) in U87 cells transfected with control sgRNA or sgRNAs targeting HNRNPH1. **E** Analysis of HNRNPH1 binding sites in the *UHRF2* pre-mRNA by eCLIP. Read density in reads per million (RPM) is shown for HNRNPH1, IgG, and input. **F** HNRNPH1 RIP followed by qRT-PCR analysis of co-purified *UHRF2* in UV-crosslinked PDGCs. **G** Diagram of *UHRF2* exon 12 – intron 12 – exon 13 sequence with sgRNAs used to generate a fragment deletion in the intron 12 sequence. HNRNPH1 binding sequence in the intronic region of the *UHRF2* is shown. **H** PCR analysis of the HNRNPH1 binding site in intron 12 of *UHRF2* in HEK293 transfected with control sgRNA or sgRNAs targeting *UHRF2* intron 12. **I** PCR analysis of exon 9-exon 10 and exon 9-exon 9a junctions for *UHRF2*.

**Supplementary Fig. 5: Silencing HNRNPH1 reduces cell division and tumorsphere formation. A** Representative microphotographs of G62 tumor sphere formation in control (up) and *HNRNPH1*-knockout cells (down). **B** qRT-PCR analysis of *HNRNPF* expression in G62 cells nucleofected with control sgRNAs or sgRNAs targeting *HNRNPH1*, followed by transfection with siRNA control or a pool of siRNAs targeting *HNRNPF*. **C** Cell viability assay of G62 PDGCs nucleofected with sgRNA control or sgRNAs targeting HNRNPH1, followed by transfection with siRNA control or a pool of siRNAs targeting *HNRNPF*. Data shown as mean ± SD of three replicates. Data were analyzed by two-way ANOVA: ****p < 0.0001. **D** Flow cytometry analysis for cell cycle distribution of U87 cells transfected with sgRNAs control or sgRNAs targeting *HNRNPH1*. Bar graphs show the quantification of cells in G1 (left), S (center), G2M (right). Data shown as mean ± SD of three replicates. Data were analyzed by unpaired t-test: **p < 0.01, ***p < 0.001. **B** Representative microphotographs of G62 control (top) and *HNRNPH1*-knockout (bottom) cells stained for Aurora B (AURKB, red) and nuclei (Hoechst33342). Cells were synchronized in the G2M phase by incubation in nocodazole. Scale bar 10 µm. **C** Representative microphotographs of G62 control cells in metaphase stained for separase (ESPL1, red) and nuclei (Hoechst33342).

**Supplementary Fig. 6: HNRNPH1 expression plays a critical role in tumor development. A** Western Blot analysis of HRNRPH1 expression in G62 cells nucleofected with sgRNAs control and two single clones grown from a pool of G62 cells nucleofected with sgRNAs targeting *HNRNPH1*. **B** Images of the brain of terminal mouse 8 implanted with *HNRNPH1*-knockout tumor. **C** Immunofluorescence of brain sections with control (top) and HNRNPH1-knockout (bottom) tumors at 11 days, stained for HNRNPH1 (red) and nuclei (Hoechst33342, blue). The white square represents the magnified area. Scale bar in whole brain microphotographs 1000 µm. The scale bar in the magnified images is 25 µm. **D and E** Sequence alignment between the *HNRNPH1* RNA isolated from Mouse 4 (**D**) and Mouse 1 (**E**) and the *HRNNPH1* RefSeq RNA. **F** Alignment of HNRNPH1 reference protein sequence with HNRNPH1 protein sequence expressed in mouse 1 tumor.

**Supplementary Table 1:** List of primers used throughout the study.

**Supplementary Table 2:** Analysis of differentially expressed genes between HNRNPH1-knockout and control U87 cells.

**Supplementary Table 3:** Analysis of differentially expressed genes between control and HNRNPH1-knockdown G62 patient-derived GBM cells.

**Supplementary Table 4:** Alternative splicing analysis comparing HNRNPH1-knockout and control U87 cells. Alternative splicing events were categorized into retained intron (RI), skipped exon (SE), alternative 5’ splice site (A5SS), mutually exclusive exons (MXE), and alternative 3’ splice site (A3SS).
