# Supplementary figures and images for: HNRNPH1 drives glioblastoma progression by regulating the splicing of cell cycle genes
Source: Cell Death Dis. 2026 Mar 24;17(1):352. doi: 10.1038/s41419-026-08576-6 (PMC13039110; doi:10.1038/s41419-026-08576-6)

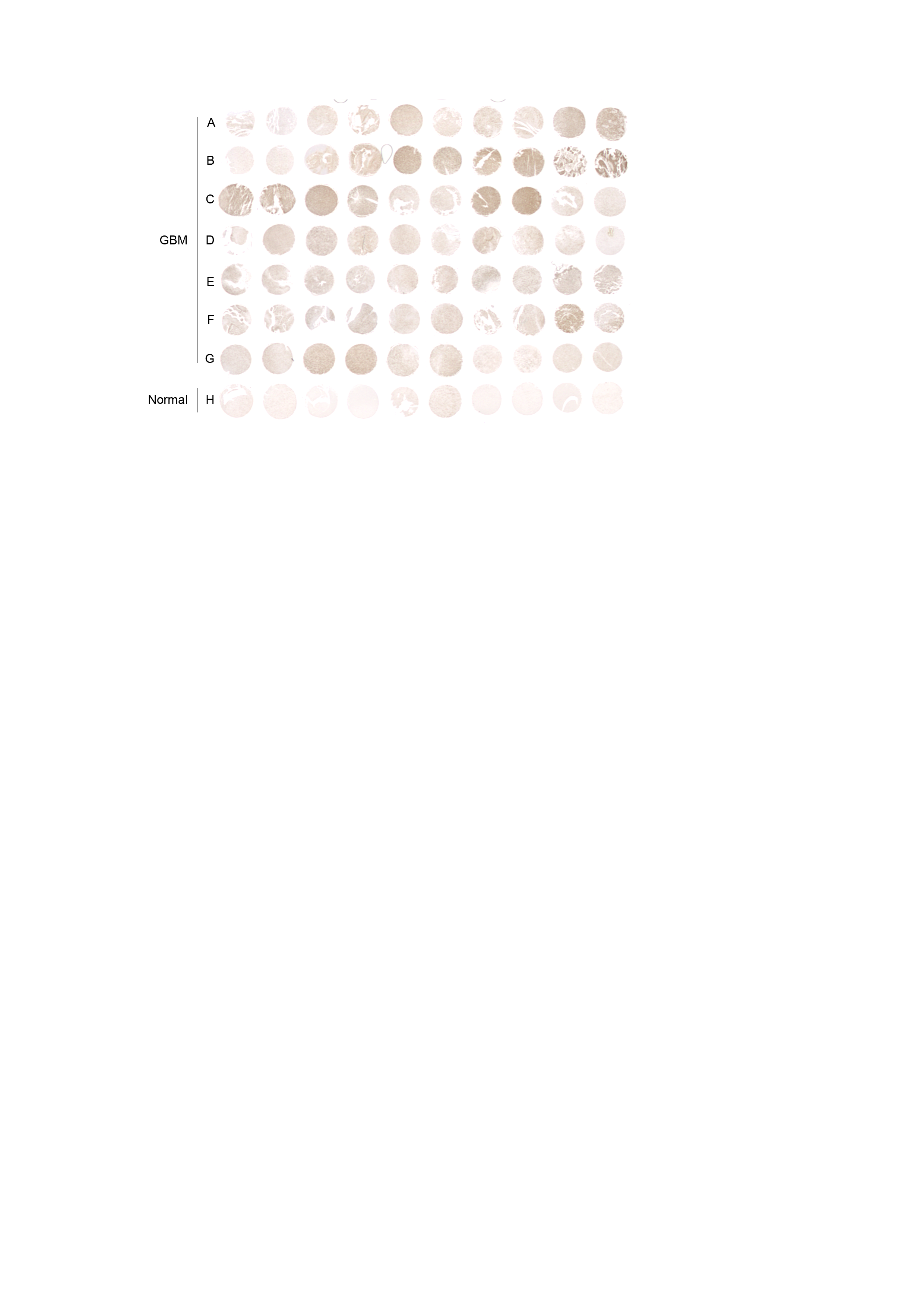

Supplement: Supplementary file 2 — Supplementary Figure 1 [file 41419_2026_8576_MOESM2_ESM.tif]

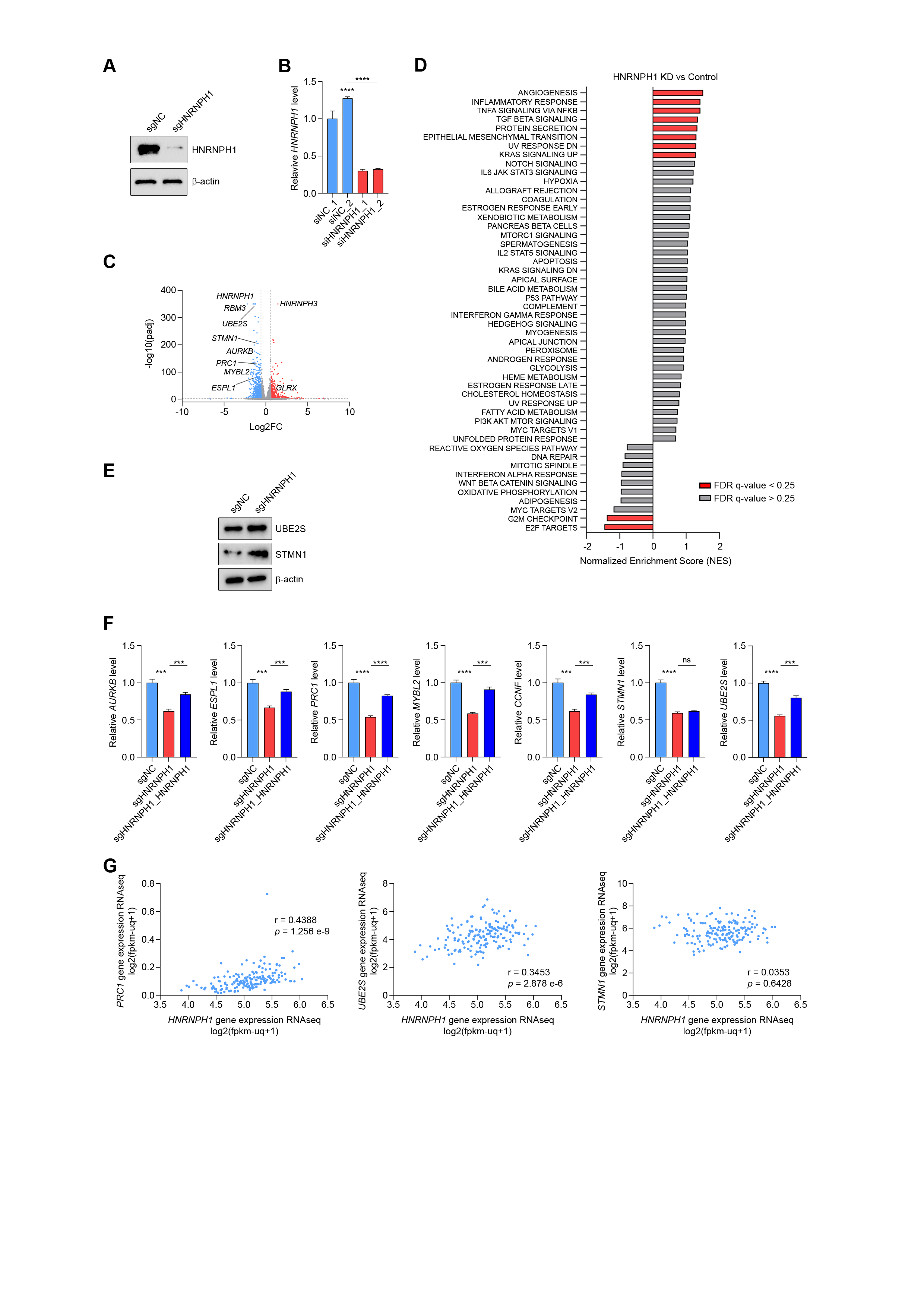

Supplement: Supplementary file 3 — Supplementary Figure 2 [file 41419_2026_8576_MOESM3_ESM.tif]

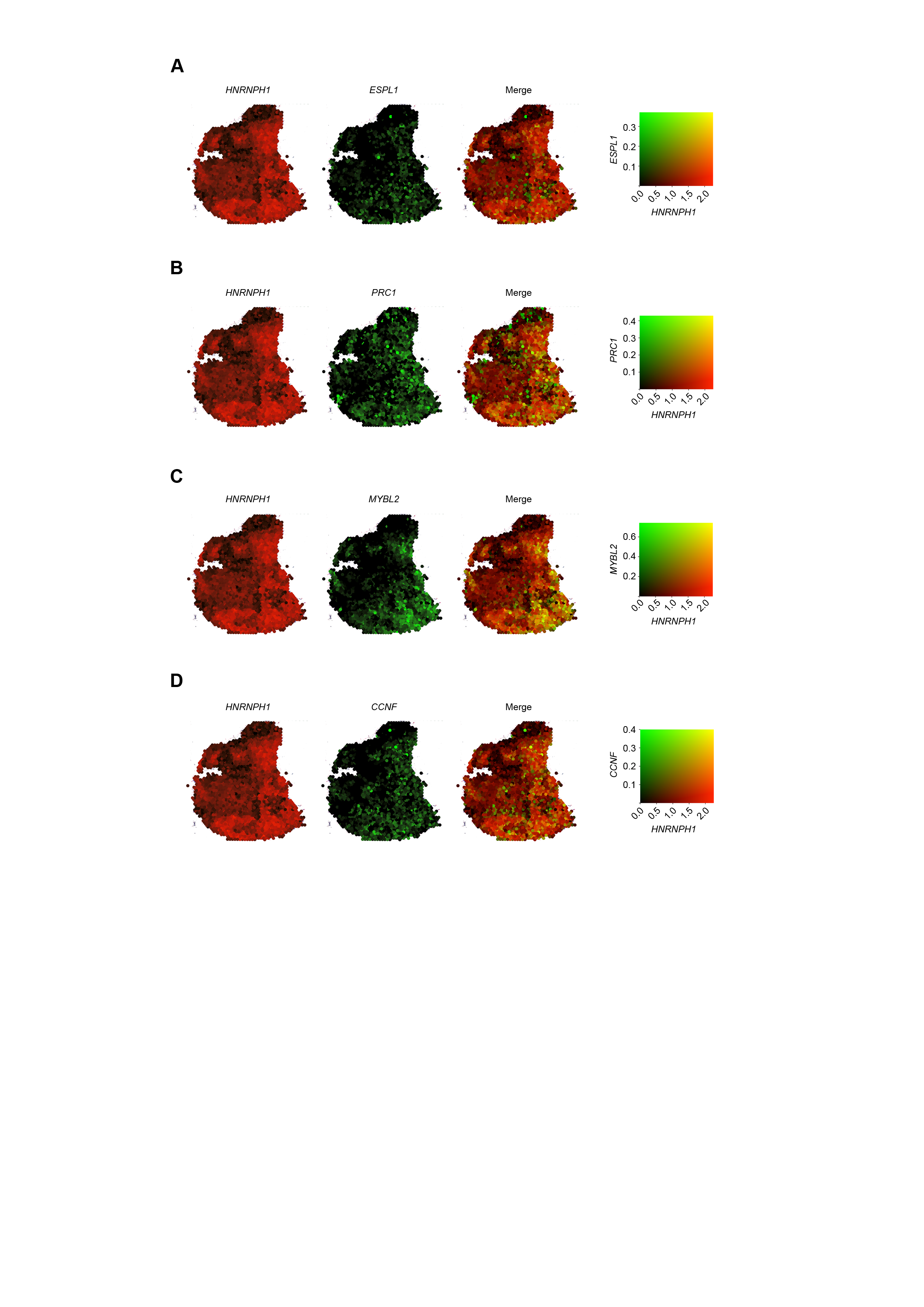

Supplement: Supplementary file 4 — Supplementary Figure 3 [file 41419_2026_8576_MOESM4_ESM.tif]

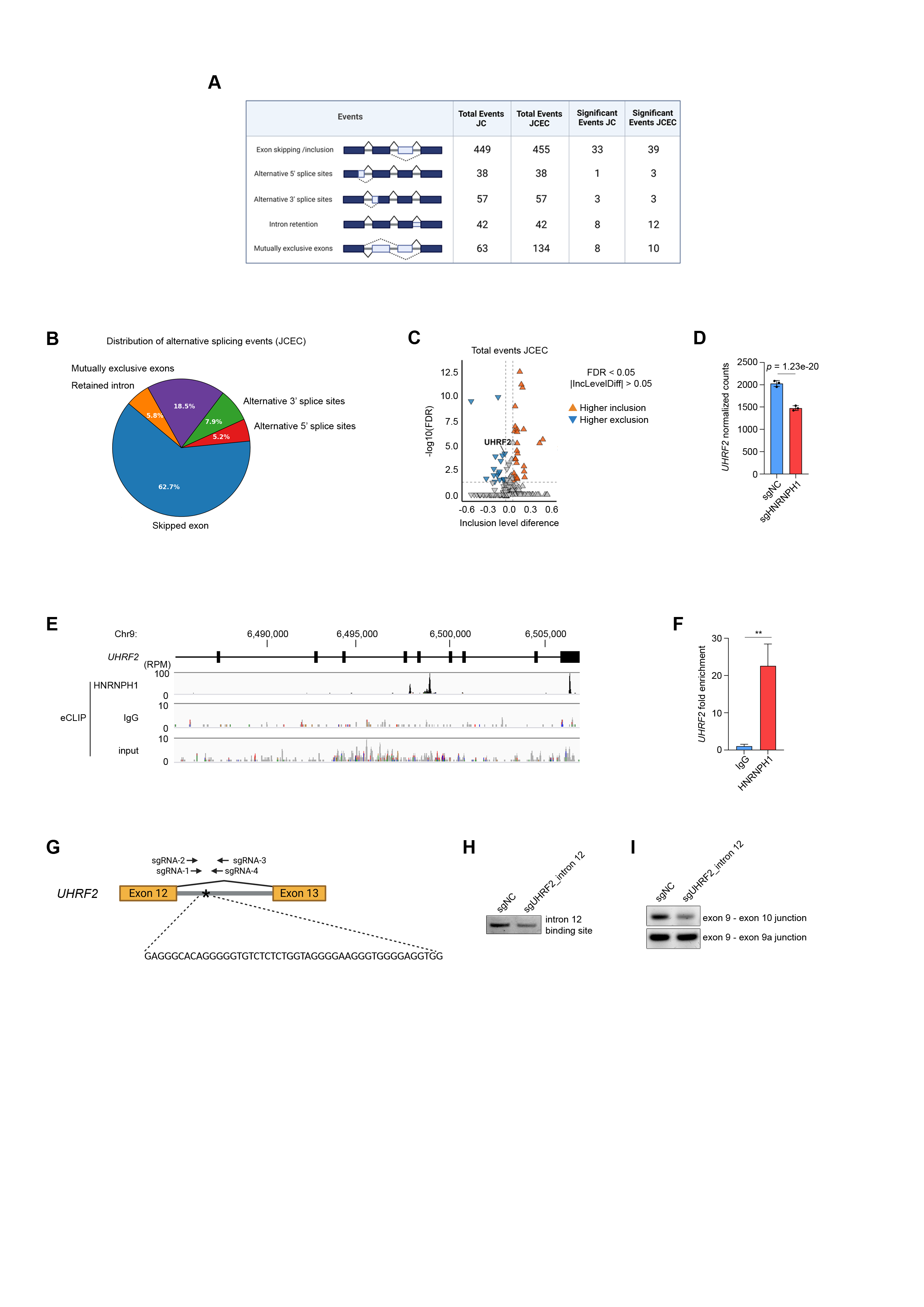

Supplement: Supplementary file 5 — Supplementary Figure 4 [file 41419_2026_8576_MOESM5_ESM.tif]

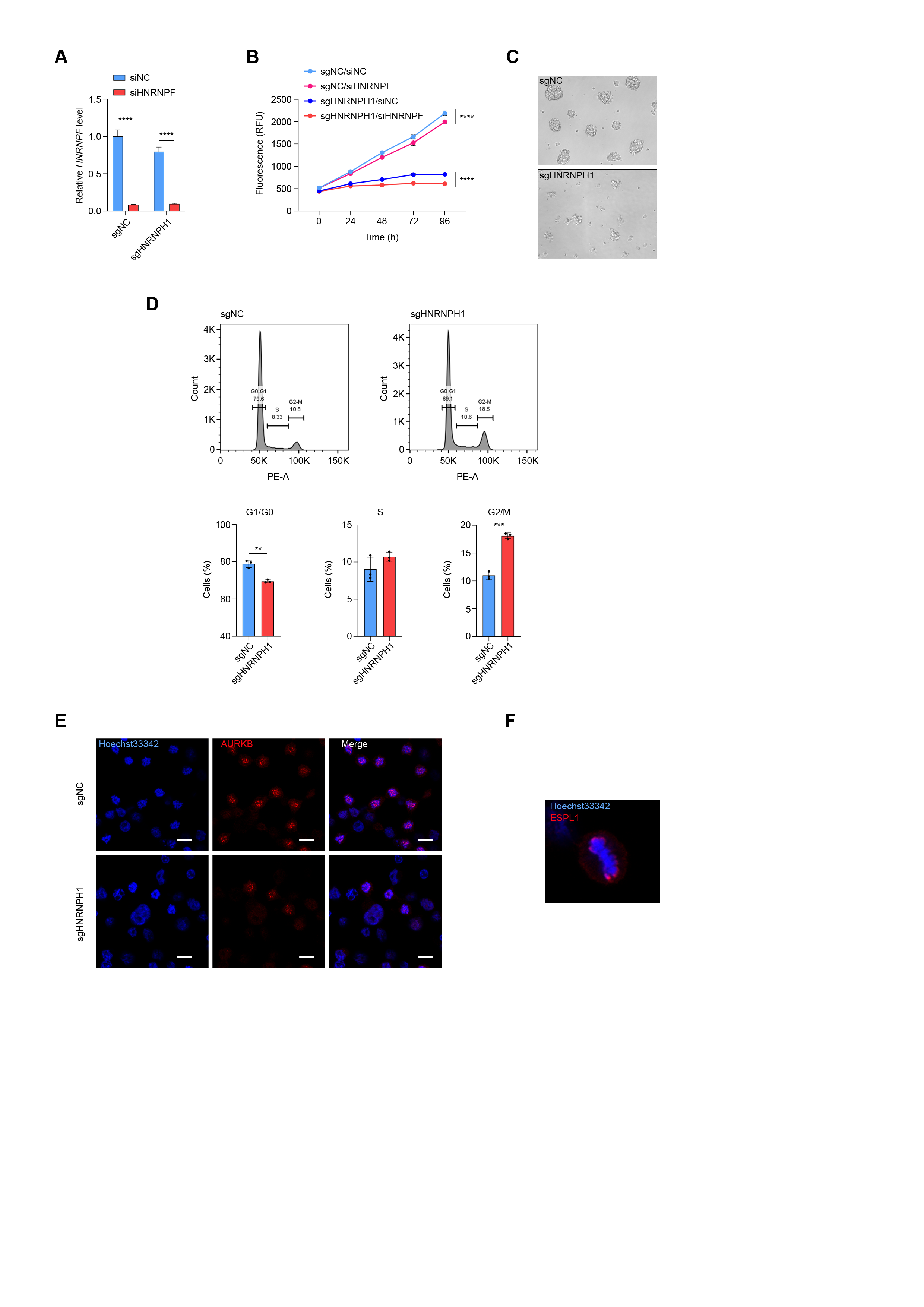

Supplement: Supplementary file 6 — Supplementary Figure 5 [file 41419_2026_8576_MOESM6_ESM.tif]

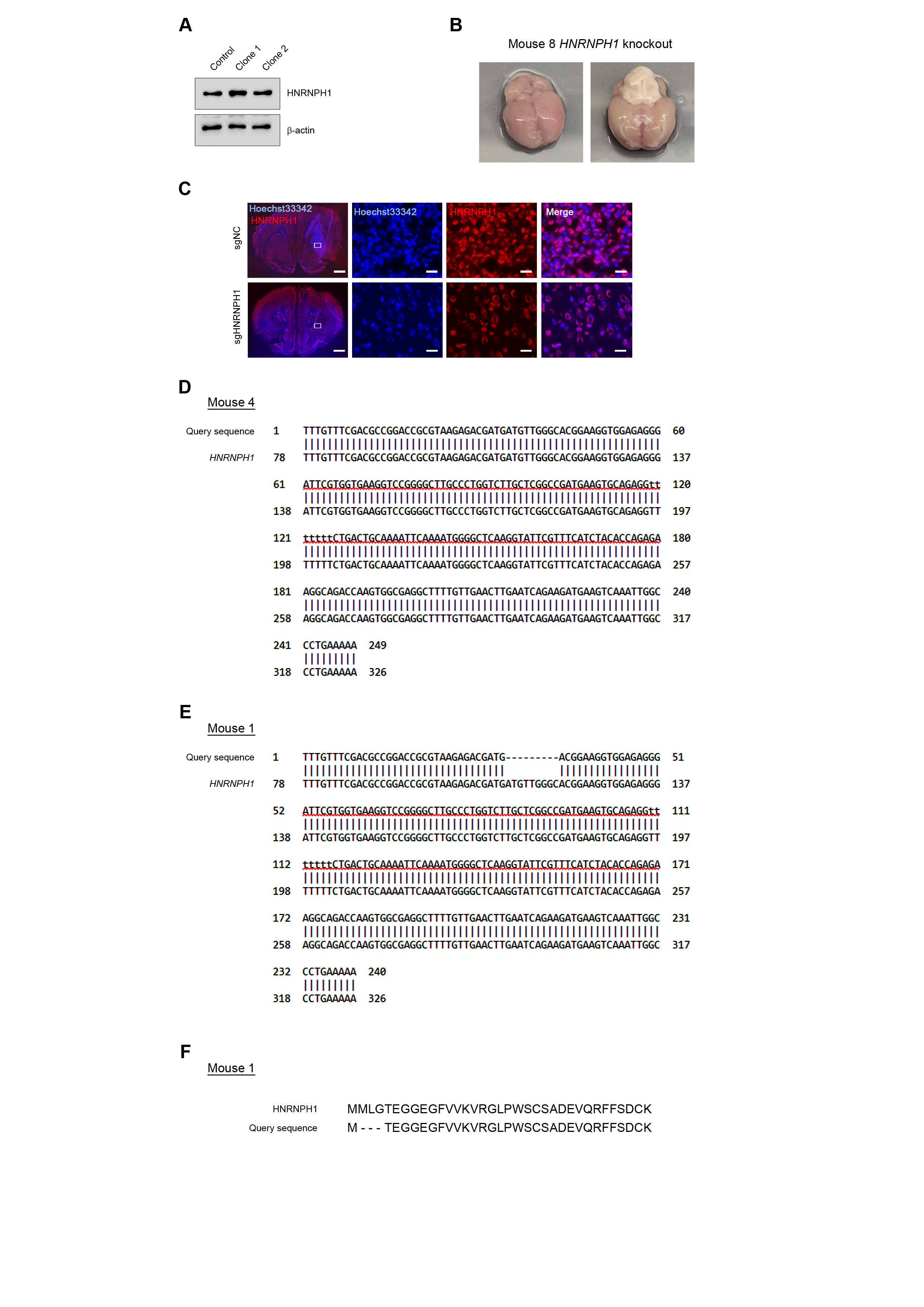

Supplement: Supplementary file 7 — Supplementary Figure 6 [file 41419_2026_8576_MOESM7_ESM.tif]
