# Supplementary material for: HNRNPH1 drives glioblastoma progression by regulating the splicing of cell cycle genes
Source: Cell Death Dis. 2026 Mar 24;17(1):352. doi: 10.1038/s41419-026-08576-6 (PMC13039110; doi:10.1038/s41419-026-08576-6)

Fig. 2I

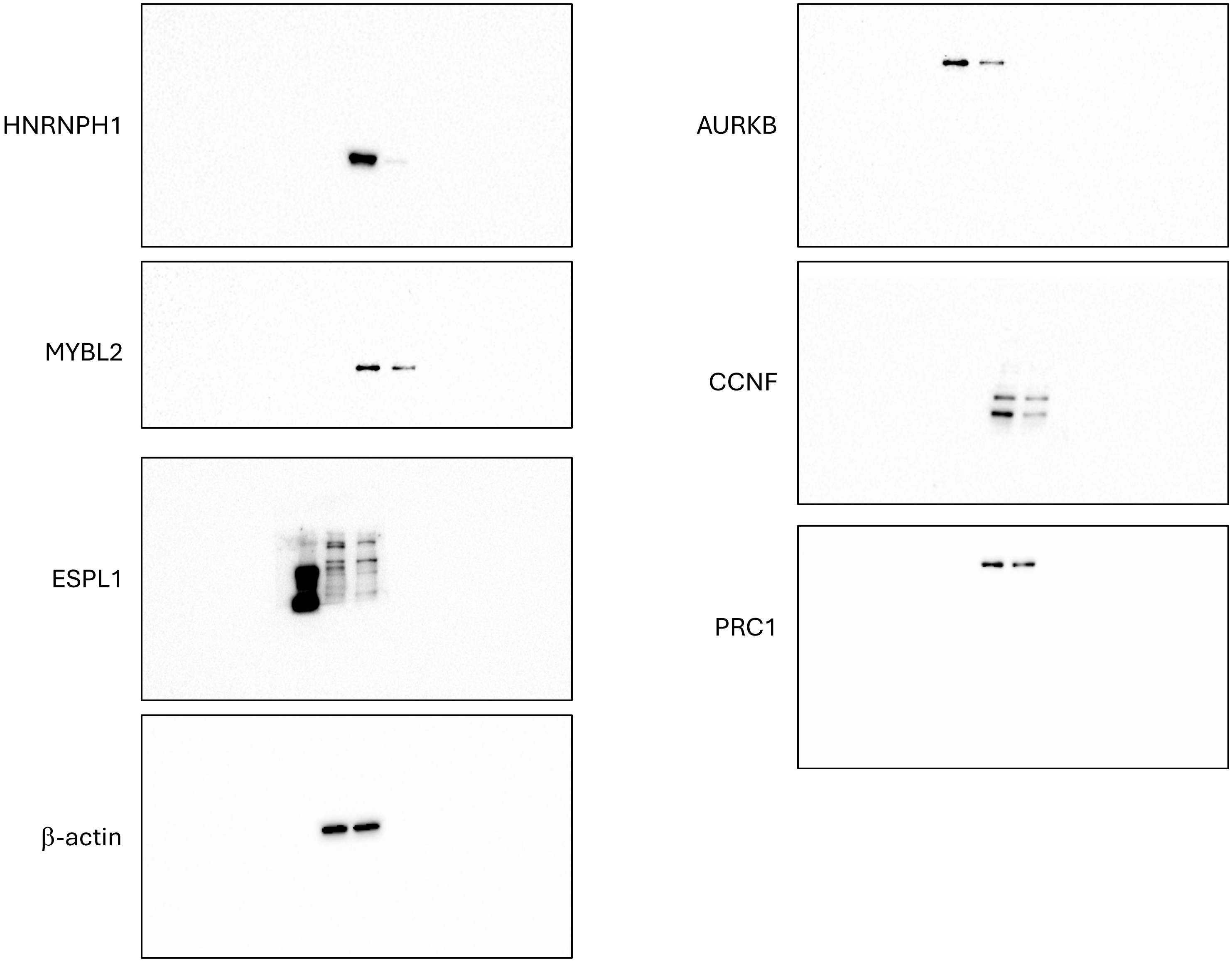

Fig. 4N

UHRF2

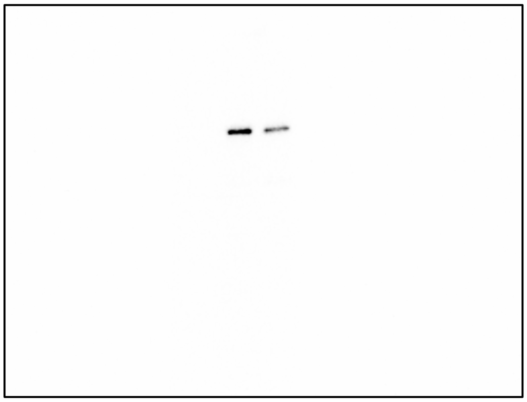

CCND1

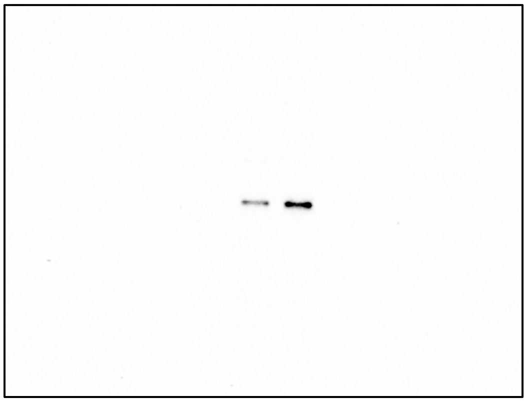

β-actin

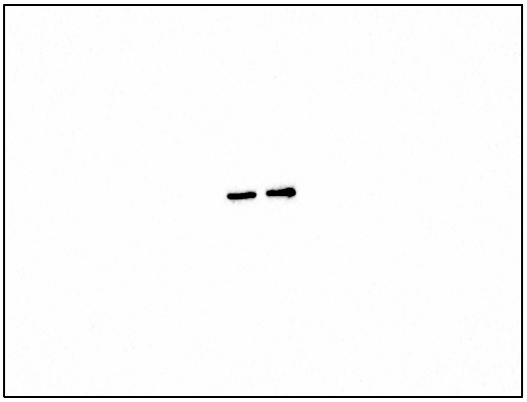

Fig. 6D

HNRNPH1

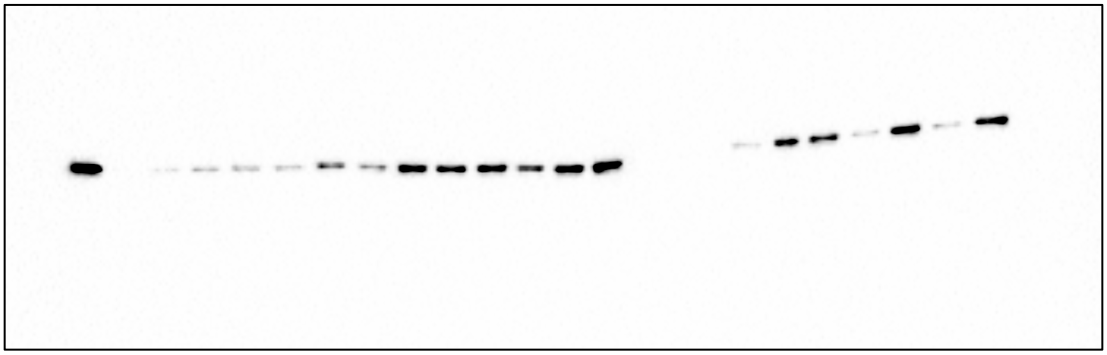

β-actin

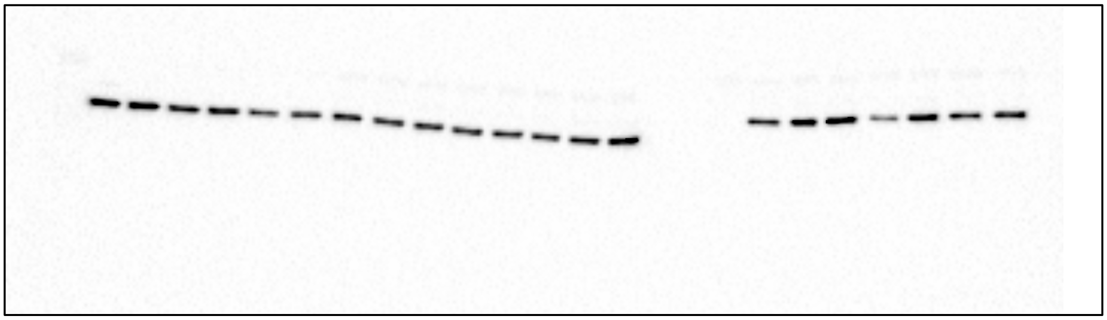

Supplementary Fig. 2A

HNRNPH1

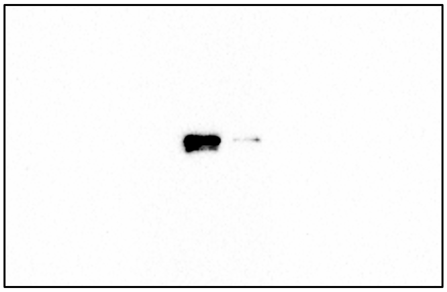

β-actin

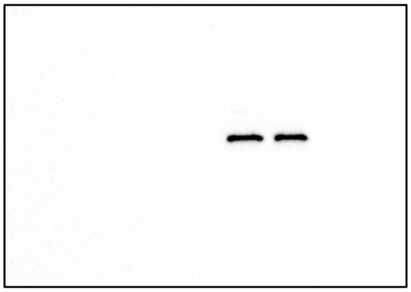

Supplementary Fig. 2E

UBE2S

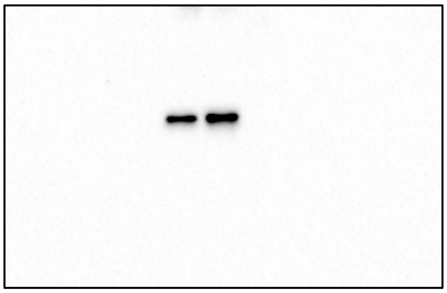

STMN1

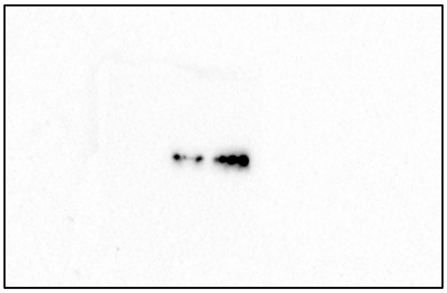

β-actin

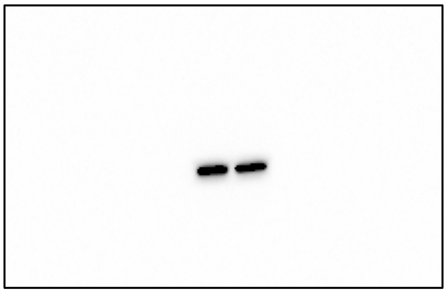

Supplementary Fig. 6A

HNRNPH1

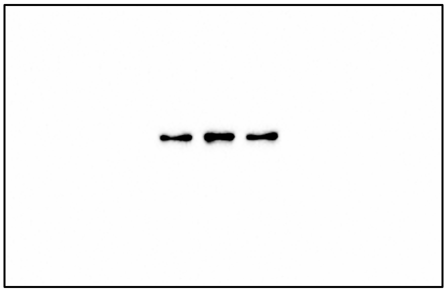

β-actin

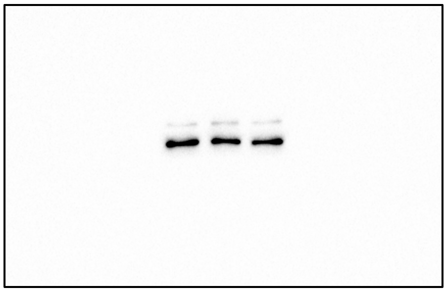

Supplement: Supplementary file 12 — Original western blots [file 41419_2026_8576_MOESM12_ESM.pdf]
